# PME-1 sensitizes glioblastoma cells to oxidative stress-induced cell death by attenuating PP2A-B55α-mediated inactivation of MAPKAPK2-RIPK1 signaling

**DOI:** 10.1038/s41420-023-01572-1

**Published:** 2023-07-27

**Authors:** Liesbeth Guffens, Rita Derua, Veerle Janssens

**Affiliations:** 1 Laboratory of Protein Phosphorylation & Proteomics, Dept. Cellular & Molecular Medicine, KU Leuven, B-3000 Leuven, Belgium; 2 KU Leuven Cancer Institute (LKI), B-3000 Leuven, Belgium; 3 SyBioMa, KU Leuven, B-3000 Leuven, Belgium

**Keywords:** Phosphorylation, Predictive markers, Methylation, Necroptosis, Stress signalling

## Abstract

Glioblastoma (GBM) is the most common primary brain tumor in adults. Current standard therapy is surgery followed by radiotherapy, with concurrent and adjuvant temozolomide chemotherapy. GBM is characterized by almost uniformly fatal outcomes, highlighting the unmet clinical need for more efficient, biomarker-guided treatments. Protein phosphatase methylesterase-1 (PME-1), a regulator of the tumor suppressive phosphatase PP2A, promotes PP2A demethylation and inactivation, and is overexpressed in 44% of GBM, associated with increased tumor grade and cellular proliferation. Here, we aimed to investigate how reactive oxygen species (ROS), a frequent by-product of radiotherapy and temozolomide chemotherapy, regulate PP2A function via its methylesterase PME-1, and how PME-1 overexpression impacts the response of GBM cells to oxidative stress. We found that in two glioblastoma cell lines, U87MG and U251MG, expression of PME-1 is positively correlated with the sensitivity of the cells to H_2_O_2_ or t-BHP-induced oxidative stress. Experiments using the irreversible pharmacologic PME-1 inhibitor, AMZ30, and different PME-1 mutants, revealed that the methylesterase function, the PP2A binding capacity, and the nuclear localization of PME-1 are all important for the sensitizing effect of PME-1 expression. Furthermore, we identified increased nuclear localization of the PP2A-B55α subunit, increased binding of PP2A-B55α to PME-1, and increased B55α-bound PP2A-C demethylation upon oxidative stress. Lastly, we uncovered increased stress-induced phosphorylation and activity of MAPKAPK2 and RIPK1 in PME-1 overexpressing U87MG cells, which caused the observed sensitization to t-BHP treatment. Our data reveal a novel role for PME-1 in oxidative stress-induced GBM cell death, regulating nuclear PP2A-B55α activity and MAPKAPK2-RIPK1 signaling. Patients with GBM tumors overexpressing PME-1, although having a worse prognosis due to increased cellular proliferation of the tumor, could actually be more responsive to oxidative stress-inducing therapies.

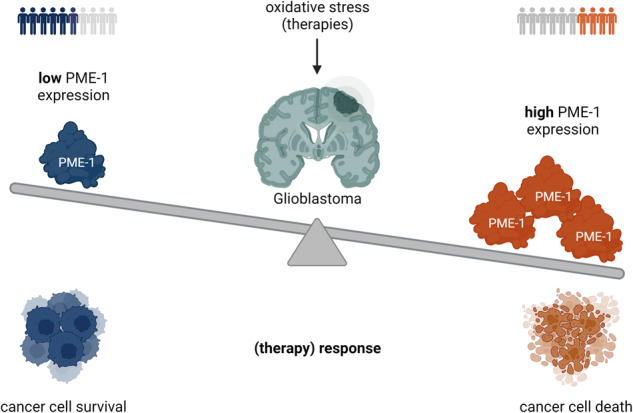

## Introduction

Reversible protein phosphorylation, catalyzed by antagonistic kinases and phosphatases, is of major importance for the regulation of cellular signaling. One of the main Ser/Thr phosphatases in the cell is protein phosphatase 2A (PP2A) [1]. PP2A represents a family of holoenzymes consisting of a core complex, composed of a catalytic subunit (PP2A-C) and a structural subunit (PP2A-A), that can be associated with one of many regulatory B subunits responsible for substrate specificity of the phosphatase [2, 3]. PP2A is involved in multiple cellular processes, including cell division and cell death [4, 5]. Recognized as a tumor suppressor [1, 6], PP2A is dysfunctional in various cancer types [7, 8]. In most cases, PP2A inactivation in cancer is achieved by non-genomic mechanisms involving overexpression of endogenous PP2A inhibitory proteins, such as cancerous inhibitor of PP2A (CIP2A) and PP2A methylesterase-1 (PME-1) [9–12]. In particular, PME-1 is frequently overexpressed in endometrial, gastric and lung cancer, hepatocellular carcinoma, and glioblastoma [13–16].

Glioblastoma (GBM) is the most common primary brain tumor in adults. It is a very aggressive cancer, associated with short survival (<10% 5-year survival) and near uniformly fatal outcome [17]. The current standard treatment is maximal surgical resection, followed by irradiation, with concurrent and adjuvant chemotherapy using temozolomide (TMZ) [18]. Due to the ineffective treatment, there is a major unmet clinical need for more efficient GBM (targeted) therapies and associated biomarkers.

In GBM, PME-1 overexpression is found in 44% of cases (*n* = 222), positively correlating with malignancy (stage), increased Ki-67 proliferation index, and increased ERK and MEK phosphorylation [16], while PME-1 inhibition restricts GBM growth [16, 19]. Moreover, PME-1 expression causes widespread kinase inhibitor resistance in GBM [19].

PME-1 has a dual role in PP2A regulation [20]. First, it mediates the reversible carboxymethylation of PP2A-C. This modification is catalyzed by S-adenosylmethionine-dependent leucine carboxyl methyltransferase 1 (LCMT1) [21] and reversed by PME-1 [22]. It differentially regulates PP2A-C assembly with the plethora of regulatory subunits that determine PP2A specificity and activity [23–25]. Enzymatic PP2A-C demethylation is dependent on a catalytic Ser residue in PME-1 (Ser156), while stable PME-1 binding to PP2A-C is not required [26, 27]. Second, PME-1 has been described to inactivate PP2A through metal eviction from the active site [28]. However, another, non-exclusive view inferred a role for PME-1 in stabilizing already inactive PP2A complexes that arise during PP2A holoenzyme biogenesis [3, 29] or immediately following its de novo biosynthesis. This function is independent on its esterase activity, but dependent on its high binding affinity for inactive PP2A-C [22, 30]. One report also suggested the importance of PME-1 methylesterase activity to protect PP2A-C from ubiquitin/proteasome degradation [31]. The stabilizing role of PME-1 may be important, as, during times of stress, the PP2A holoenzyme can disassemble [32], increasing the risk of uncontrolled activity of free PP2A-C. This infers the existence of potential PME-1 regulation mechanisms under cellular stress conditions.

Specifically, in GBM, PME-1 expression or inhibition of PP2A can indeed sensitize cells to different stresses. For instance, pharmacologic inhibition of PP2A with LB100 enhanced the effects of DNA-damaging agents TMZ and doxorubicin [33] and increased the radiosensitivity of GBM cells [34]. In GBM stem cells, PME-1 knockdown inhibited hypoxia-induced cell death [35], and glucose deprivation-mediated cell death in U251MG cells was shown to be dependent on PME-1 and on stress-induced PP2A demethylation and inactivation [36]. Thus, a picture emerges, in which PP2A and its cellular modulator PME-1 are not only in control of GBM growth, but also of the specific response of GBM cells to a large variety of cytotoxic stress factors. However, the molecular mechanisms mediating this PME-1-mediated regulation of PP2A in stressed cells, and regulation of PME-1 itself, under these circumstances, have mostly remained unclear.

Here, we investigated the role of differential PME-1 expression on the sensitivity of GBM cells to oxidative stress, a stress that is often associated with radiotherapy and TMZ chemotherapy, the current standard treatments for GBM.

## Results

### PME-1 expression sensitizes GBM cells to oxidative stress

**Fig. 1:**
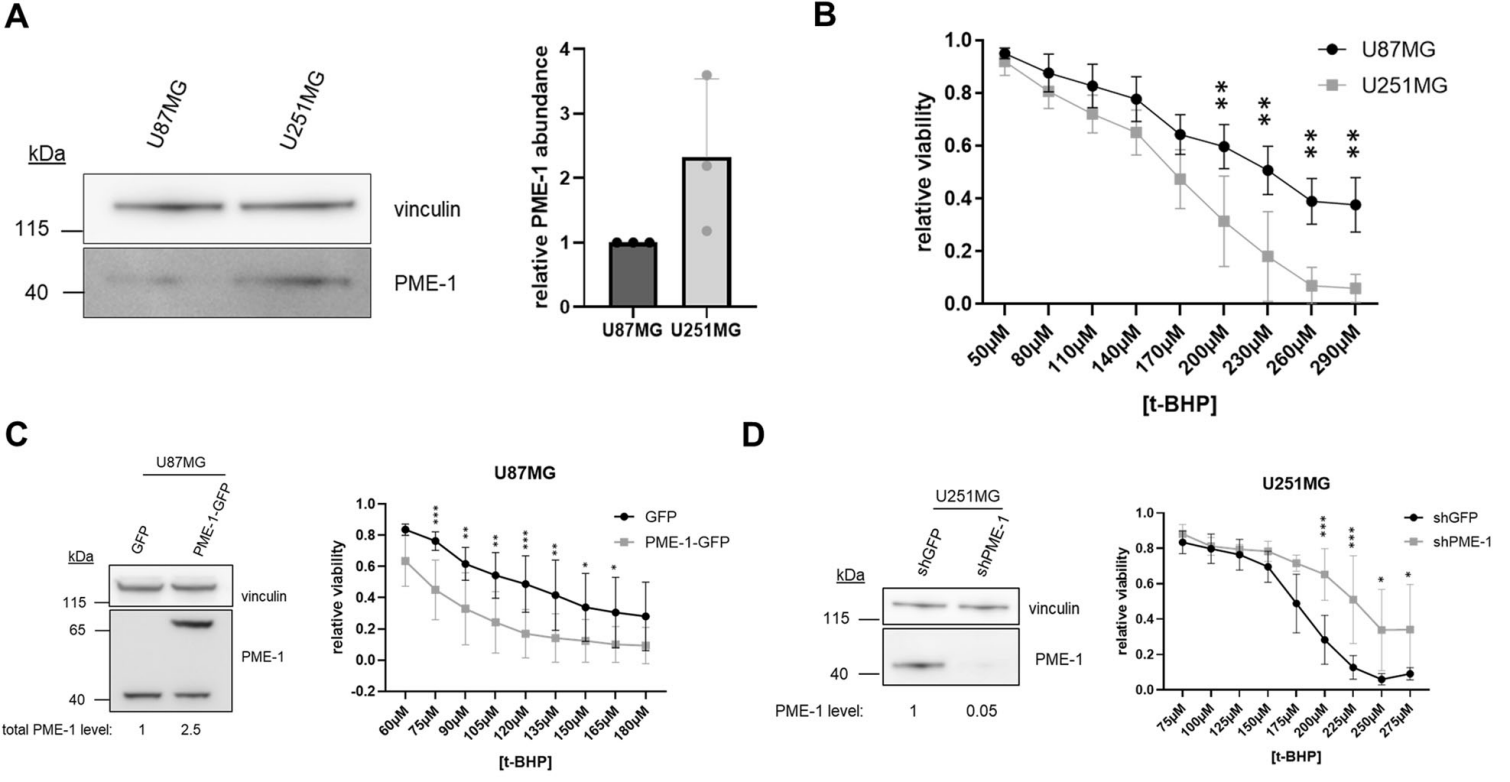
PME-1 expression sensitizes GBM cells to oxidative stress. **A** Endogenous PME-1 levels in U87MG and U251MG cells were determined and quantified using Western blotting (*N* = 3, two-tailed *t*-test); a representative blot is shown. **B** Sensitivity of U87MG and U251MG cells to 16 h t-BHP treatment (different concentrations) was determined using MTT (*N* = 3, two-way ANOVA). **C** Validation of stable PME-1-GFP overexpression in U87MG cells using Western blotting (left). Quantification below the blots refers to ‘total’ PME-1 levels ( = endogenous PME-1 + PME-1-GFP). The sensitivity of U87MG PME-1-GFP cells to 16 h treatment with indicated concentrations of t-BHP was determined using MTT (*N* = 4, two-way ANOVA) (right). **D** Validation of stable PME-1 knockdown in U251MG cells using Western blotting (left). The sensitivity of U251MG shPME-1 cells to 16 h treatment with indicated concentrations of t-BHP was determined using MTT (*N* = 5, two-way ANOVA) (right).

It is currently not known how PME-1 expression affects the response of GBM cells to oxidative stress. Therefore, we first assessed endogenous PME-1 expression in two glioblastoma cell lines, U87MG and U251MG, and checked their sensitivity to oxidative stress (Fig. 1A, B). We induced oxidative stress by treating cells with H_2_O_2_, or for phenotypic experiments, with the more stable H_2_O_2_ homolog t-BHP. After 16 h of treatment with t-BHP, U251MG cells, which express more PME-1 than U87MG cells (Fig. 1A), appear to be significantly more sensitive to t-BHP-induced cell death, as measured in MTT assay (Fig. 1B).

To further elucidate the importance of PME-1 in the oxidative stress response in an isogenic cell context, we chose to increase PME-1 expression in the PME-1 low-expressing cell line and to reduce PME-1 expression in the PME-1 high-expressing cell line. We generated a stable PME-1-GFP overexpressing U87MG cell line and a PME-1 depleted U251MG cell line using a PME-1 3’UTR-targeting shRNA. The resulting PME-1 expression levels were verified by immunoblotting (Fig. 1C, D). Again, we observed increased sensitivity of the U87MG PME-1-GFP overexpressing cells compared to the GFP overexpressing control (Fig. 1C), and conversely, found increased resistance of U251MG shPME-1 cells compared to the shGFP control (Fig. 1D). These data underscore the sensitizing role of PME-1 in the oxidative stress response of two independent GBM cell lines.

### Methylesterase activity, PP2A-C binding capacity, and nuclear localization of PME-1 all contribute to the sensitizing effect of PME-1 overexpression to oxidative stress

**Fig. 2:**
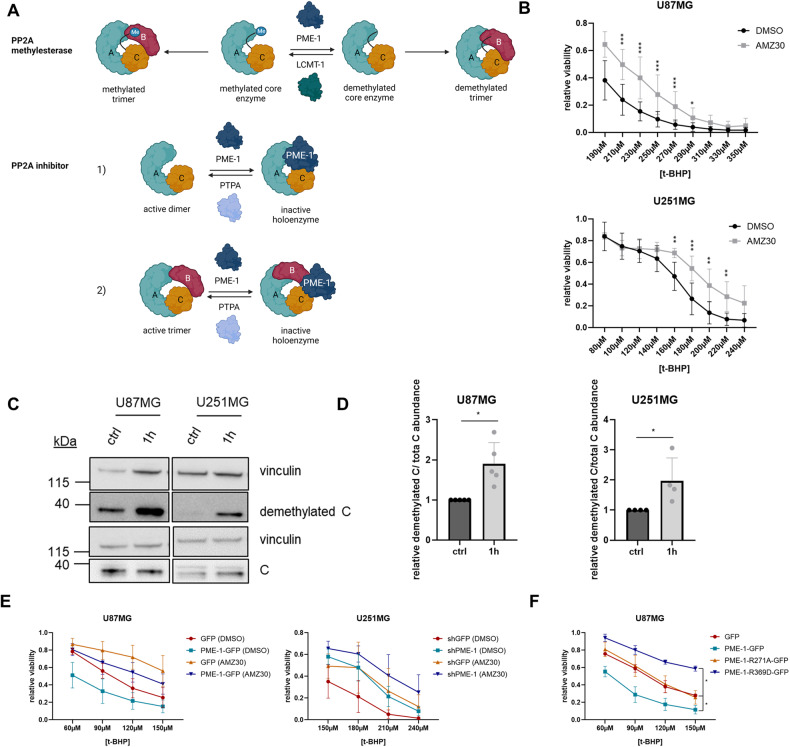
Methylesterase activity, PP2A-C binding capacity, and nuclear localization of PME-1 all contribute to the PME-1-dependent decrease in cell viability upon oxidative stress. **A** Schematic representation of both possibly interconnected functions of PME-1 as PP2A methylesterase and PP2A inhibitor (created with BioRender.com). **B** Sensitivity of U87MG and U251MG cells to 16 h t-BHP treatment (different concentrations) with or without 1 h pretreatment with 20 μM AMZ30 was determined using MTT (*N* = 4, two-way ANOVA). **C** Demethylated PP2A-C and total PP2A-C levels in U87MG and U251MG cells were determined after 1 h treatment with 1 mM H_2_O_2_ using Western blotting (representative blots). **D** Quantification of demethylated PP2A-C shown in (**C**) (normalized to total PP2A-C) (*N* = 5 (U87MG), *N* = 4 (U251MG), two-tailed *t*-test). **E** Sensitivity of U87MG PME-1-GFP and U251MG shPME-1 cells to 16 h t-BHP treatment (different concentrations) with or without pretreatment with 20 μM AMZ30 was determined using MTT (*N* = 4 (U87MG), *N* = 6 (U251MG), statistical significance is visualized in Fig. S1A). **F** Sensitivity of U87MG cells expressing PME-1-GFP (WT), PME-1 R271A-GFP (NLS mutant), or PME-1 R369D-GFP (PP2A-C binding mutant) to 16 h treatment with different concentrations of t-BHP was determined using MTT (*N* = 3, two-way ANOVA).

As explained, PME-1 plays a dual role in PP2A regulation (Fig. 2A): as the enzyme responsible for PP2A-C demethylation, and as a PP2A-C stabilizer/inhibitor during PP2A holoenzyme biogenesis or following holoenzyme disassembly [20]. Another characteristic of PME-1 is its predominant nuclear localization due to the presence of a functional nuclear localization signal (NLS) [26].

To assess whether the methylesterase activity of PME-1 contributed to the sensitizing effect of PME-1 to oxidative stress, we used the catalytic PME-1 inhibitor AMZ30, which irreversibly inhibits PME-1 demethylating activity [37]. Interestingly, in the presence of AMZ30, the resistance of both U87MG and U251MG cells to t-BHP increased (Fig. 2B). Furthermore, in the absence of AMZ30, demethylated PP2A-C levels increased in response to H_2_O_2_ treatment in both cell lines (Fig. 2C, D), suggesting increased methylesterase activity of PME-1 under these conditions. These results indicate that the demethylating activity of PME-1 plays an important role in the oxidative stress response and may contribute to the sensitizing effect of PME-1 overexpression.

To further strengthen this hypothesis, we pretreated U87MG PME-1-GFP and U251MG shPME-1 cells with AMZ30 and measured their sensitivity to t-BHP treatment (Fig. 2E and Fig. S1A (statistics)). We found that the sensitizing effect of PME-1 overexpression in the U87MG cells is abolished by pretreatment with AMZ30. Similar results were seen in U251MG cells, in which the increased sensitivity of the shGFP cells disappeared after pretreatment with AMZ30 (Fig. 2E). Thus, the demethylating activity of PME-1 is crucial for the sensitizing effect of PME-1 overexpression in response to oxidative stress.

To assess the importance of the PP2A binding capacity and the nuclear localization of PME-1, we generated two PME-1 mutants devoid of either of these characteristics and (over)expressed them in the PME-1-low U87MG cell line, in order to see if they could sensitize the cells to the same extent as wild type PME-1 to t-BHP-induced oxidative stress. Neither the PP2A-C non-binding mutant (PME-1 R369D) [28, 38], nor the PME-1 NLS mutant (PME-1 R271A) [26] could mimic the sensitizing effect of PME-1 WT overexpression (Fig. 2F). On the contrary: the PME-1 R369D mutant even made the cells more resistant to oxidative stress compared to the control cell lines (Fig. 2F), suggestive for a dominant-negative effect.

We conclude that PME-1’s role in response to oxidative stress is multifaceted and that its methylesterase activity, nuclear localization, and capacity to bind to PP2A-C are all necessary factors contributing to its stress sensitizing effect.

### Oxidative stress promotes B55α nuclear localization, PP2A-B55α binding to PME-1 and B55α-bound PP2A-C demethylation

**Fig. 3:**
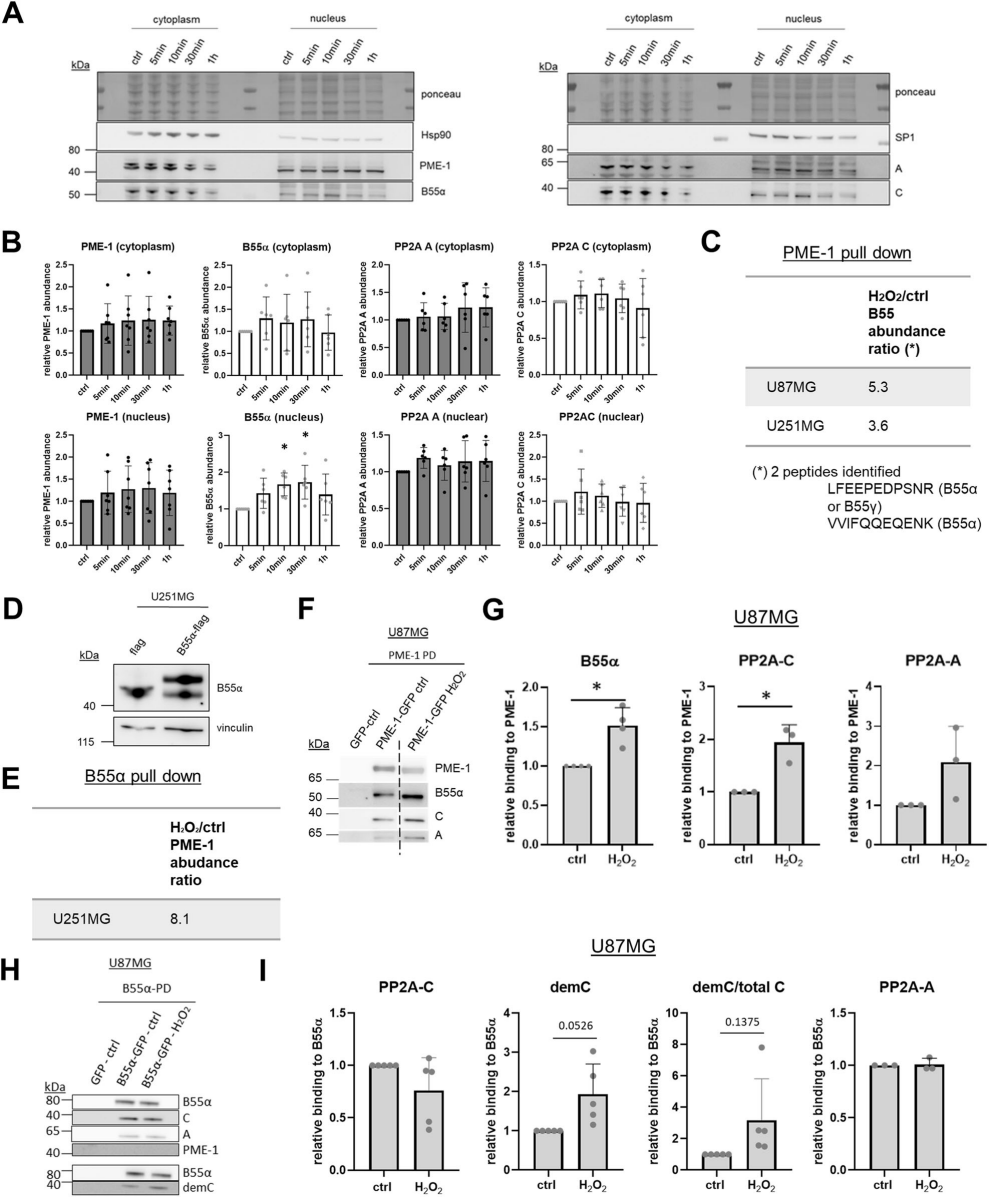
Oxidative stress promotes B55α nuclear localization, PP2A-B55α binding to PME-1, and B55α-bound PP2A-C demethylation. **A** Western blot of cytoplasmic (Hsp90 marker) and nuclear (SP1 marker) lysates of U87MG cells were treated with 1 mM H_2_O_2_ for the indicated duration. **B** Quantification of nuclear and cytoplasmic B55α and PME-1 levels (relative to ponceau) shown in **A** (*N* = 5 or 6, one-way ANOVA). **C** B55 protein abundance ratio in the PME-1-GFP interactome as determined by LC-MS/MS with or without a 10 min treatment with 10 mM H_2_O_2_. **D** Validation of stable B55α-flag expressing U251MG cells using Western blotting. **E** PME-1 protein abundance ratio in the B55α-flag interactome as determined by LC-MS/MS with or without a 10 min treatment with 2.5 mM H_2_O_2_. **F** B55α, PP2A-C, and PP2A-A subunit binding to PME-1-GFP in U87MG cells was determined after 1 h treatment with 1 mM H_2_O_2_ by GFP pull-down followed by Western blotting (non-cropped blots are shown in Fig. S2A). **G** Quantification of B55α, PP2A-C, and PP2A-A subunit binding to PME-1-GFP shown in **F** (*N* = 3 or 4, one-way ANOVA). **H** PP2A-C, demethylated PP2A-C and PP2A-A binding to B55α-GFP in U87MG cells was determined after 1 h treatment with 1 mM H_2_O_2_ by GFP pull-down followed by Western blotting. **I** Quantification of PP2A-C, demethylated PP2A-C, demethylated PP2A-C/total PP2A-C, and PP2A-A binding to B55α-GFP shown in **H** (*N* = 3 or 5, one-way ANOVA).

As Tang et al. recently described a redistribution of PME-1 to the cytoplasm in H_2_O_2_-treated human primary dermal fibroblasts [39], we prepared nuclear and cytoplasmic extracts of stressed (1 mM H_2_O_2_) and unstressed U87MG cells to examine potential changes in the subcellular localization of PME-1 upon oxidative stress. Interestingly, we observed no redistribution of PME-1 to the cytoplasm, but found an increase in the level of the PP2A regulatory B55α subunit in the nucleus during oxidative stress treatment (Fig. 3A, B). PP2A-C and -A subunits did not show an obvious translocation (Fig. 3A, B).

Furthermore, when we assessed potential, oxidative stress-induced changes in the PME-1 interactome by an affinity purification (AP)-LC-MS/MS-based approach in PME-1-GFP overexpressing cells, we found an approximately fivefold and fourfold increased binding of B55 relative to PME-1-GFP in stressed versus untreated U87MG and U251MG cells, respectively (Fig. 3C). Two different B55 peptides were identified: one unique for the B55α isoform and one common to B55α and B55γ. The B55 family of PP2A B subunits consists of four isoforms (α, β, γ, δ) that rely on PP2A-C methylation for their assembly into trimeric PP2A-B55 holoenzymes [24, 40] and regulate different cellular signaling cascades [41]. We further focused on the increased interaction of B55α with PME-1, although we cannot exclude that B55γ may also be involved in the oxidative stress response in GBM cells.

In a reciprocal AP-LC-MS/MS experiment executed in B55α-flag overexpressing U251MG cells (Fig. 3D), PME-1 showed approximately eightfold increased binding relative to B55α-flag after H_2_O_2_-induced oxidative stress (Fig. 3E). To further validate and extend these data, we performed immunoblot experiments using PME-1-GFP pull-downs isolated from H_2_O_2_-treated U87MG cells, and did not only find a clear increase in B55α, but also in PP2A-C and a trend towards increased PP2A-A binding to PME-1 (Fig. 3F, G). Similar results were seen upon treatment with t-BHP (Fig. S2B, C).

Additionally, we performed GFP pull-downs on lysates isolated from H_2_O_2_-treated U87MG cells, transiently transfected with B55α-GFP (Fig. 3H, I). Although binding of PP2A-C and PP2A-A subunits to B55α was not altered upon H_2_O_2_ treatment, bound PP2A-C appeared more demethylated in stressed conditions, consistent with the observed increase in PME-1 binding to B55α by the MS approach (Fig. 3E). The latter could however only be occasionally reproduced by Western blotting (Fig. S2D, E), as in most experiments, PME-1 appeared below the detection limit in B55α-GFP pull-downs (Fig. 3H).

Together, our data suggest increased oxidative stress-induced nuclear localization of PP2A-B55α, followed by an increased interaction with PME-1, and increased PP2A-C demethylation within the PME-1-PP2A-B55α complex.

### PME-1 overexpression induces increased MAPKAPK2 phosphorylation/activity during oxidative stress, which subsequently causes the observed cell death sensitization

**Fig. 4:**
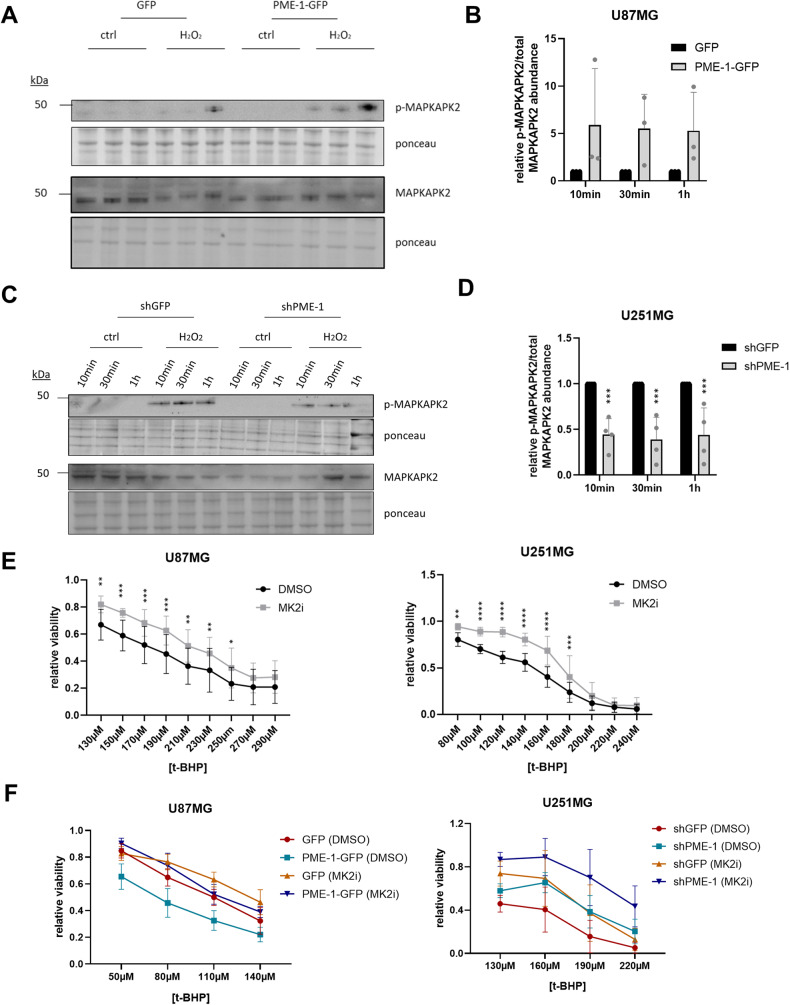
PME-1 overexpression promotes increased MAPKAPK2 phosphorylation during oxidative stress, which is causally involved in the observed increase in cell death. **A** Representative Western blots of phospho-MAPKAPK2 and total MAPKAPK2 levels in U87MG PME-1-GFP cells after treatment with 2.5 mM H_2_O_2_ for the indicated duration. **B** Quantification of phospho-MAPKAPK2/total MAPKAPK2 shown in panel **A** (*N* = 3, two-way ANOVA). **C** Representative Western blots of phospho-MAPKAPK2 and total MAPKAPK2 levels in U251MG shPME-1 cells after treatment with 2.5 mM H_2_O_2_ for the indicated duration. **D** Quantification of phospho-MAPKAPK2/total MAPKAPK2 shown in panel **C** (*N* = 3, two-way ANOVA). **E** Sensitivity of U87MG and U251MG cells to 16 h t-BHP treatment (different concentrations) with or without 1 h pretreatment with 10 μM MK2i was determined using MTT (*N* = 3 (U87MG), *N* = 4 (U251MG), two-way ANOVA). **F** Sensitivity of U87MG PME-1-GFP and U251MG shPME-1 to 16 h t-BHP treatment (different concentrations) with or without 1 h pretreatment with 10 μM MK2i was determined using MTT (*N* = 4 (U87MG), *N* = 6 (U251MG), statistical significance is shown in Fig. S1B).

The p38 mitogen-activated protein kinase (p38/MAPK) pathway is known to be involved in many cellular stress responses [42]. Interestingly, we observed a clear increase in the phosphorylation of MAPKAPK2 (Thr334), a downstream substrate of p38, in H_2_O_2_-treated U87MG PME-1-GFP cells, while phosphorylation of MAPKAPK2 was decreased in H_2_O_2_-treated U251MG shPME-1 cells compared to their respective controls (Fig. 4A–D). Phospho-p38 and phospho-MAPKAPK2 form a productive signaling complex in the nucleus that quickly translocates to the cytoplasm upon MAPKAPK2 activation [43]. Although the inactivation of MAPKAPK2 by specific Ser/Thr phosphatases has remained largely elusive [43], MAPKAPK2 has been suggested as a direct PP2A substrate [44]. As Thr334 resides in a TP phosphorylation motif (PQT^334^P), it would be an excellent substrate for dephosphorylation by PP2A-B55 holoenzymes [45, 46].

To assess whether increased phosphorylation, and thus kinase activity, of MAPKAPK2 is causally related to decreased viability upon oxidative stress in our GBM cell models, we next pretreated U87MG and U251MG cells with 10 μM PF-364402 (MAPKAPK2 inhibitor, MK2i) before exposing them to t-BHP (Fig. 4E). In both cell lines, we found that inhibition of MAPKAPK2 activity with MK2i indeed resulted in increased viability upon exposure to t-BHP. More importantly, we repeated this experiment in U87MG PME-1-GFP and U251MG shPME-1 cells (Fig. 4F and Fig. S1B (statistics)), and found that the increased sensitivity of the U87MG PME-1-GFP cells to oxidative stress was entirely abolished by pretreatment with MK2i, while the increased sensitivity of the U251MG shGFP cells compared to the shPME-1 cells likewise disappeared in the presence of MK2i. We conclude that the increased phosphorylation/activity of MAPKAPK2 observed in PME-1-high-expressing GBM cells is, therefore, causally linked to the increased sensitivity of these cells to oxidative stress.

### PME-1 overexpression induces increased RIPK1 phosphorylation/activity during oxidative stress, which subsequently causes the observed cell death sensitization

**Fig. 5:**
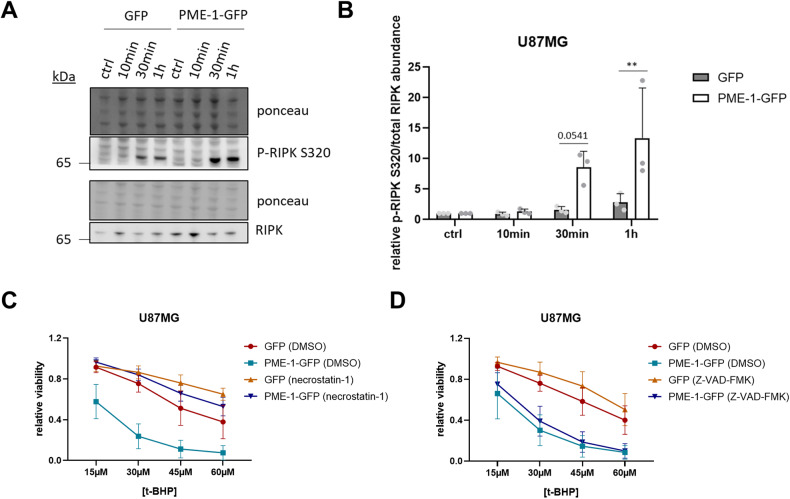
Increased sensitivity of PME-1 overexpressing GBM cells to oxidative stress correlates with increased RIPK1 phosphorylation, and is inhibited by necrostatin-1 but not by a pan-caspase inhibitor. **A** Representative Western blots of phospho-RIPK1 and total RIPK1 levels in U87MG PME-1-GFP cells after treatment with 2.5 mM H_2_O_2_ for the indicated duration. **B** Quantification of phospho-RIPK1/total RIPK1 shown in panel **A** (*N* = 3, two-way ANOVA). **C** Sensitivity of U87MG PME-1-GFP cells to 16 h t-BHP treatment (different concentrations) with or without 8 h pretreatment with 100 μM necrostatin-1, as determined by MTT (*N* = 4, statistical significance is shown in Fig. S3A). **D** Sensitivity of U87MG PME-1-GFP cells to 16 h t-BHP treatment (different concentrations) with or without 8 h pretreatment with 100 μM Z-VAD-FMK, as determined by MTT (*N* = 4, statistical significance is shown in Fig. S3B).

The necroptosis-inducing kinase RIPK1 is a known phosphorylation target of MAPKAPK2 upon TNFR activation [47]. To uncover if MAPKAPK2 also regulates RIPK1 upon oxidative stress in our GBM cell lines, we assessed potential changes in RIPK1 Ser320 phosphorylation upon treatment with H_2_O_2_. Not only did we observe RIPK1 phosphorylation of Ser320 upon H_2_O_2_ treatment, we also found increased phospho-RIPK1 levels in U87MG PME-1-GFP cells as opposed to the U87MG GFP control cells (Fig. 5A, B). Moreover, pretreatment of the cells with 100 μM necrostatin-1 (a RIPK1 inhibitor) [48] selectively abolished the sensitizing effect of increased PME-1 expression (Fig. 5C and Fig. S3A (statistics)), while pretreatment with 100 μM Z-VAD-FMK (a pan-caspase inhibitor) had no effect on t-BHP-induced cell death (Fig. 5D and Fig. S3B (statistics)). Thus, upon oxidative stress, PME-1 overexpression facilitates a form of cell death that is RIPK1 activity-dependent and does not rely on caspase activity (i.e., “necroptosis”).

**Fig. 6:**
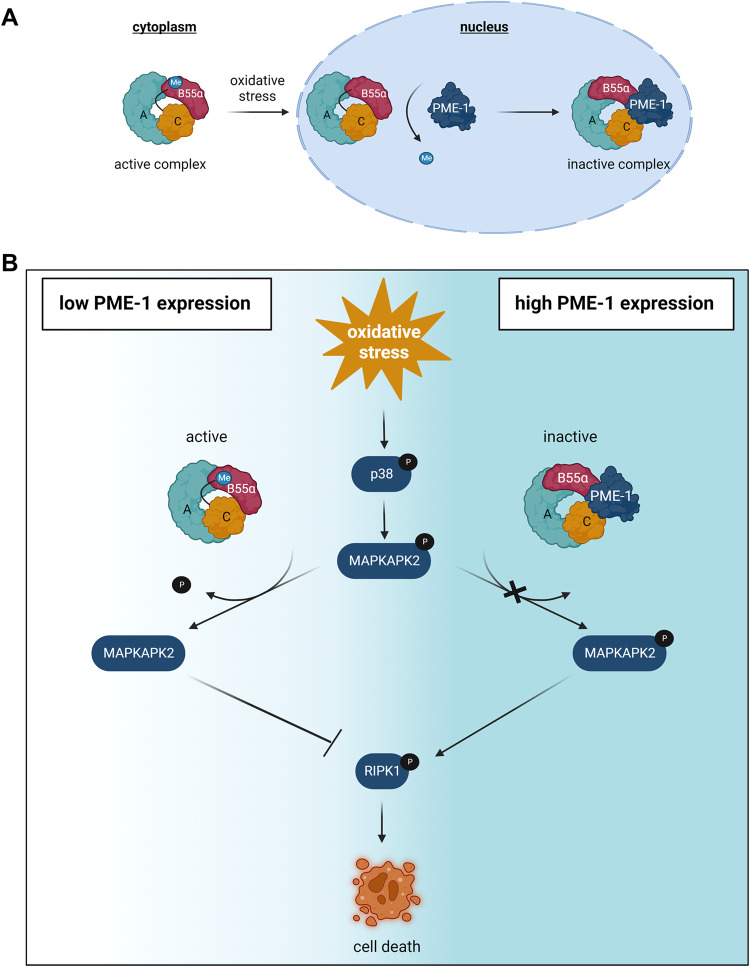
Model of the sensitizing effect of PME-1 to oxidative stress-induced cell death in GBM. **A** Visual representation of oxidative stress-induced PP2A-B55α binding to PME-1 and subsequent PP2A-C demethylation and inactivation in the nucleus (created with BioRender.com). **B** Model of the PP2A-B55α-inactivating function of PME-1 that subsequently controls activation of the MAPKAPK2-RIPK1 pathway and induction of RIPK1-dependent cell death (necroptosis) (created with BioRender.com).

We conclude that, upon oxidative stress, PME-1-mediated inhibition of nuclear PP2A-B55α promotes increased MAPKAPK2 Thr334 phosphorylation and activity in PME-1-high-expressing GBM cells. This increased MAPKAPK2 activity subsequently induces higher levels of RIPK1 Ser320 phosphorylation and activity, which eventually causes increased GBM cell death (Fig. 6).

## Discussion

PP2A dysregulation and the emerging therapeutic options of pharmacologic PP2A modulation have recently received increasing attention from the brain oncology community [49, 50]. Specifically in GBM, overexpression of cellular PP2A inhibitors, such as CIP2A [51–53], ARPP-19 [9], or PME-1 [16, 19], constitutes one of the major PP2A inhibitory mechanisms. PME-1 was found to be overexpressed in 44% of GBM, correlating with increased malignancy and proliferation [16]. Although patients with PME-1-high tumors were reported to be more resistant to kinase inhibitor therapies [19], the current study indicates these patients might be more responsive to oxidative stress-inducing therapies. While the oncogenic role of PME-1 was explained by its activating effect on MEK-ERK signaling [16], the sensitizing role of PME-1 to oxidative stress-induced cell death could be attributed to its activating effect on MAPKAPK2-RIPK1 signaling, via the regulation of nuclear PP2A-B55α holoenzymes.

In cells, reactive oxygen species (ROS), such as superoxide anions, hydrogen peroxide, and hydroxyl radicals, can be formed during the metabolism of oxygen and in response to different xenobiotics or chemicals. In addition, reactive nitrogen species (RNS) can be formed through chemical reactions with NO. Oxidative stress occurs when there is an imbalance between ROS production and ROS removal by antioxidants [54]. Cancer cells are, in general, more sensitive to oxidative stress due to their higher metabolic rate [55]. This initiated an interest in oxidative therapies, which are aiming at further increasing ROS levels to induce cancer cell death [56]. Here, we observed an increased sensitivity of PME-1 overexpressing GBM cells to t-BHP or H_2_O_2_-induced oxidative stress. Therefore, PME-1 expression could potentially serve as a marker to stratify GBM patients when testing oxidative therapies, or to stratify patients in non/semi/good-response groups for current standard therapies (radiotherapy and TMZ chemotherapy), which both induce oxidative stress as well [57]. However, it remains to be formally determined whether PME-1 expression will indeed affect the outcome of these DNA-damaging therapies, as predicted by our current study.

The impact of our findings on human disease may be broader than just the treatment of GBM or other cancer types in which PME-1 is frequently overexpressed [13–16]. Indeed, a similar response might be triggered in normal brain cells upon oxidative stress. This should be studied more in detail and could potentially broaden the therapeutic relevance of our findings. For example, PME-1 overexpression is commonly observed in brain samples from patients with tauopathies, such as progressive supranuclear palsy (PSP) and Alzheimer’s disease (AD) [58]. Oxidative stress generally increases upon aging, and high levels of ROS are commonly observed in patients with neurodegenerative diseases [59]. Therefore, inhibiting PME-1 by AMZ30, or inhibiting PME-1-controlled MAPKAPK2-RIPK1 signaling by MK2i or necrostatin-1, could potentially limit an increase in neural cell death caused by high oxidative stress levels in patients with PSP or AD. Moreover, AMZ30 may not only prevent neural cell death, it might also limit the formation of tau aggregates by preventing PME-1-mediated inhibition of PP2A-B55α–catalyzed tau dephosphorylation [60].

PP2A regulation during oxidative stress has been repeatedly demonstrated, although results from published studies were not always consistent, and could be explained by context- and holoenzyme-dependent differences [61, 62]. However, most studies demonstrated PP2A inhibition by ROS or RNS in diverse cell contexts and through multiple mechanisms [63–67]. ROS can result in direct oxidation of at least two cysteine residues in PP2A-C [64], as well as in direct glutathionylation of PP2A-C [63]. Both modifications are reversible by the addition of reducing agents, and inhibit PP2A activity in vitro and in cells [68]. RNS-mediated inhibition of PP2A has been shown to occur by direct tyrosine nitration of PP2A-C [69], or of the B56δ subunit—in the latter case, resulting in its failure to be incorporated into a PP2A holoenzyme [67]. More relevant to our current study, ROS-induced PP2A inhibition has frequently been correlated with PP2A-C demethylation and increased apoptosis, e.g., in neurons [66], embryonic kidney [70], and cancer cells [65]. Here, we mechanistically extended these findings by showing for the first time that oxidative stress cannot only lead to PP2A-C demethylation in general, but to a specific demethylation (and inactivation) of the PP2A-B55α holoenzyme in the nucleus, and to a specific form of cell death dependent on RIPK1 activity (necroptosis). Although the observed increase in PME-1-PP2A-B55α binding is likely attributable to an increased presence of PP2A-B55α in the nucleus (Fig. 3A, B), we cannot entirely exclude that potential post-translational modifications of B55α or PME-1, such as e.g., the previously described PME-1 Ser15 phosphorylation [71], might further facilitate their interaction. However, although we specifically investigated this possibility, we failed to identify any H_2_O_2_-induced changes in PME-1 or B55α phosphorylation in our MS-based assays (data not shown). It also remains to be determined how the increased nuclear presence of B55α in the stressed cells can be explained (translocation, stabilization), although a translocation of PP2A-B55 in the opposite direction (from the nucleus into the cytoplasm) has been observed by others in DNA-damaged cells [72]. Although we could not detect a clear concomitant stress-induced increase of PP2A-C or -A in the nucleus, we presume that this is still the case, since B55α is not stable as a monomer [24, 41] and the translocating (B55α-bound) fraction of PP2A-C and -A might just be too small to be discriminated from the non-translocating PP2A-C and -A pool (bound to other B-type subunits) in a Western blot assay. In any case, the increased nuclear B55α presence upon oxidative stress could serve as a physiologic way to restrict excessive stress-induced signaling pathways in the nucleus, and, thereby, to prevent induction of cell death. Only in the pathologic case of PME-1 overexpression, this nuclear function of PP2A-B55α would specifically be attenuated, and stress-induced cell death would be promoted. As explained before, this is obviously of interest in cancer therapy, but is not desirable in the aging brain.

While PME-1 relocalization to the cytoplasm after H_2_O_2_ treatment has been described in primary dermal fibroblasts [39], we could not observe a redistribution of PME-1 to the cytoplasm in GBM cells. Also, the PME-1 NLS mutant, which localizes to the cytoplasm [26], failed to sensitize GBM cells to oxidative stress-induced cell death, further underscoring that the site of PME-1 function and regulation of PP2A-B55α is not the cytoplasm, but the nucleus.

The binding of trimeric PP2A-B55α to PME-1 has been observed before by three independent groups [30, 38, 73], with Longin et al. initially reporting on an inactive, PME-1-bound PP2A-B55 holoenzyme purified de novo from a porcine brain that served as a good substrate in an in vitro methylation turnover assay [30]. The latter implied that the PP2A-B55 holoenzyme is a good PME-1 substrate, although several crystallographic studies [28, 60, 74, 75] originally suggested significant steric hindrance of PME-1 binding to the PP2A AC core by the regulatory B subunits. Recently, however, using a cryo-EM approach, Li et al. revealed that large structural shifts in both the holoenzyme and PME-1 do allow PME-1 to bind to PP2A-B56 trimers and block the substrate-binding groove [76]. They also discovered that two distinct disordered regions within PME-1 mediate its interaction with PP2A-B56 and with PP2A-B55 [76]. Thus, it is entirely conceivable that increased binding of PME-1 to PP2A-B55α can indeed result in PP2A-B55α demethylation and catalytic inactivation, as observed in our study.

The p38 pathway is known to be important in transducing oxidative stress signals [42]. MAPKAPK2 is a direct downstream target of p38 in the nucleus, which, on its turn, can phosphorylate several cellular substrates, including RIPK1 [47, 77, 78]. In our GBM models, we found that in response to oxidative stress PME-1 overexpression, by inhibiting nuclear PP2A-B55α activity, increased the phosphorylation (and activation) of MAPKAPK2 Thr334, to promote subsequent RIPK1 Ser320 phosphorylation, and finally, cell death. As PP2A-B55 trimers preferentially dephosphorylate proline-directed phospho-threonines [46], we speculate that the p38 phosphorylation site of MAPKAPK2 (Thr344) is the likely target of PP2A-B55α. Although several cellular substrates of PP2A-B55α have already been identified [41], this would be the first time MAPKAPK2 is suggested as a direct PP2A-B55α target, whose dephosphorylation is moreover controlled by PME-1. In addition, PP2A has been identified as a possible (direct or indirect) upstream phosphatase of RIPK1 in glucose-deprived conditions [36]. Although certain papers pointed to a cell death inhibiting effect of RIPK1 Ser320 phosphorylation downstream of death receptor engagement [47, 77, 78], others have shown that RIPK1 Ser320 and Ser166 phosphorylation mediate necroptosis under oxidative stress conditions [79], very much in line with our current findings.

We anticipate that the PP2A-modulating role of PME-1 in different forms of stress-induced cell death may be much broader than currently anticipated, and certainly deserves to be further investigated in other cell and stress contexts.

## Materials and methods

### Cloning and site-directed mutagenesis

PME-1 (encoded by *PPME1*) was cloned into pEGFP-N1 (Clontech). B55α (encoded by *PPP2R2A*) was cloned into pEGFP-N1 and 3xFLAG CMV10 vector (Sigma). PME-1 mutants (in pEGFP-N1) were generated by PCR-based site-directed mutagenesis (primers in Table S1). Mutations were confirmed by sequencing (LGC Genomics). Lentiviral expression vectors (pLKO.1) for *PPME1* 3’UTR targeting (shPME-1, target sequence: AACATCGAGCTCTGTTGTAA) and control GFP-targeting shRNA (shGFP) were generated as described [80].

### Cell culture and transfection

Human GBM cell lines U87MG (ATCC, #HTB-14) and U251MG (ECACC, #09063001) were cultured at 37 °C and 5% CO_2_ in DMEM (Sigma, #D6546) supplemented with 10% heat-inactivated fetal bovine serum (Sigma, #F7524), 2 mM l-glutamine (Sigma, #G7513), and 100 units/mL penicillin and 100 mg/mL streptomycin (Sigma, #P0781). Transfections were performed with lipofectamine 3000 (Thermo Fisher, #L3000001). All cell experiments were performed at passage number <30. Cultures were regularly tested for *Mycoplasma* contamination (Venor™ GeM, Minerva Biolabs, #11-1050).

### Generation of stable cell lines with altered PME-1 or B55α expression

Stable PME-1-GFP and B55α-flag overexpressing cells were generated by transfection of pEGFP-N1 (containing *PPME1* cDNA) or 3xFLAG CMV10 vector (containing *PPP2R2A* cDNA) with lipofectamine 3000 (Thermo Fisher, #L3000001) and subsequent Geneticin (G418) selection at 400 μg/ml. Polyclonal cell populations were frozen and used in all subsequent experiments.

For the generation of lentiviral particles, TurboFect (Thermo Fisher, #R0533) was used to transfect 500 ng lentiviral expression vector (pLKO.1), 450 ng packaging vector (pCMV-deltaR 8.91), and 50 ng enveloping vector (pMD.G-VSVG) in HEK293T cells. Twenty-four hours and 48 h after transfection, lentiviral particles were harvested and filtered through a 0.45 μm syringe filter. For transductions, U87MG and U251MG cells were incubated with different virus titers for 24 and 48 h, and selected with puromycin (1.25 μg/ml) for 1 week. Polyclonal cell populations were frozen for further use.

### Chemical inhibitors and compounds

Tert-butyl hydroperoxide (t-BHP, #A13926) was purchased from Alfa Aesar, MAPKAPK2 inhibitor PF-364402 (#PZ0188) and H_2_O_2_ (#H1009) from Sigma, AMZ30 (#539695) from Millipore, Z-VAD-FMK (#S8102) from Bio-Connect and necrostatin-1 (#T1547) from Tebubio.

### Protein extraction, immunoprecipitation, and Western blotting

Cells were rinsed with PBS, lysed with NET-lysis buffer (50 mM Tris-HCl, pH 7.4, 1% NP-40, 15 mM EDTA, and 150 mM NaCl) supplemented with protease inhibitor cocktail (Roche) and phosphatase inhibitors (PhosStop, Roche) and centrifuged for 15 min at 13,000 × *g*. For PME-1-GFP or B55α-flag/pull-down followed by Western blot analysis, phosphatase inhibitors were omitted from the lysis buffer.

For pull-down experiments, 300 μl lysate was incubated at 4 °C for 1 h with 500 μl NENT100 (20 mM Tris-HCl, pH 7.4, 1% NP-40, 15 mM EDTA, and 100 mM NaCl) containing 1 mg/mL bovine serum albumin (BSA), and 40 μl prewashed GFP-trap-A beads (ChromoTek) or anti-flag mAb conjugated sepharose beads (FLAG M2 affinity gel, Sigma) on a rotating wheel. Beads were washed three times with 0.5 mL NENT150 (containing 150 mM NaCl). Bound proteins were eluted in 2x NuPage sample buffer (Invitrogen) and boiled at 95 °C for 10 min, prior to loading on a denaturing SDS-PAGE gel (4–12% Bis-Tris Midi, Invitrogen). Proteins were transferred to a nitrocellulose membrane (GE Healthcare) by wet blotting at 100 V for 40 min. Membranes were blocked in 5% BSA in TBS/0. 1% Tween-20 for 1 h at room temperature and incubated with primary antibody overnight at 4 °C. Membranes were washed three times with TBS-T and incubated with HRP-conjugated secondary antibodies (Dako) for 1 h at room temperature. After a final wash with TBS-T, membranes were developed using Western Bright ECL (Advansta) on the ImageQuant LAS 4000 scanner (GE Healthcare). Densitometric analysis was performed using Image Studio lite software.

### Antibodies

Antibodies were obtained from the indicated suppliers: anti-PME-1 (Santa Cruz Biotechnology, #sc-25278), PP2A-C (Cell Signaling Technology, #2038), vinculin (Sigma-Aldrich, #V9131), B55α (Cell Signaling Technology, #5689), PP2A-A (mouse monoclonal, gift from Prof. S. Dilworth), MAPKAPK2 (Cell Signaling Technology, #3042), phospho-MAPKAPK2 (Cell Signaling Technology, #3007), demethylated PP2A-C (Sigma-Aldrich, #05-577), SP1 (Cell Signaling Technology, #9389), Hsp90 (Cell Signaling Technology, #4877), RIPK1 (Cell Signaling Technology, #3493), and phospho-RIPK1 Ser320 (Cell Signaling Technology, #58274).

### Nuclear and cytoplasmic extraction

Subcellular fractionation was performed with NE-PER nuclear and cytoplasmic extraction kit (Thermo Fisher, #7833), according to the manufacturer’s instructions.

### MTT proliferation assay

Cells were seeded in 96-well plates at a density of 3,000 cells/well and treated for 16 h with different concentrations of t-BHP. After 16 h, the medium was replaced by a medium containing 0.5 mg/mL Thiazolyl Blue Tetrazolium bromide (MTT; Alfa Aeser, #L11939.06). After 4-h-incubation, the medium was exchanged for DMSO to dissolve the formazan crystals. Absorbance was measured at 550 nm in a multichannel spectrophotometer.

### Affinity purification (AP)—LC/MS-MS analysis

Immunoprecipitates of PME-1-GFP or B55α-flag were washed three times with 0.5 mL NENT150 (containing 150 mM NaCl), followed by three times with 0.5 ml NH_4_HCO_3_ (AMBIC) (200 mM), and subjected to an overnight on-bead digestion at 37 °C in the presence of 50 mM AMBIC, 5% CH_3_CN, 0.01% ProteaseMax (Promega), and 1 μg trypsin (Pierce). The resulting peptides were desalted by Micro Spin Tips (Harvard Apparatus) and subjected to high-resolution LC-MS/MS, using an Ultimate 3000 Nano Ultra High Pressure Chromatography system interfaced with an Orbitrap Elite Hybrid Ion Trap-Orbitrap (Q Exactive) mass spectrometer via an EASY-spray (C-18, 15 cm) column (Thermo Fisher Scientific). Peptides were identified by MASCOT (Matrix Science) using UniProt *Homo sapiens* (173,330 sequences) as a database. Progenesis software (Nonlinear Dynamics) was used for relative quantification of peptides, and Proteome Discoverer 2.2 software (Thermo Fisher Scientific) for peptide validation (FDR <1%) using Percolator node. Protein abundances were normalized according to the protein abundance of the bait in different conditions.

### Statistics

Data were represented as mean plus standard deviation (SD), unless described otherwise. Statistical tests, as indicated in figure legends, were performed using GraphPad Prism 8.0.1. Significance was represented as *, **, ***, and **** representing *p* values of ≤0.05, ≤0.01, ≤0.001, and ≤0.0001, respectively. “*N*” denotes the number of biological replicates.

## Supplementary information

Supp Material - Tables and Figures

Original Data File

## Data Availability

The data that support the findings of this study are available from the corresponding author upon reasonable request.
