# Supplementary material for: PME-1 sensitizes glioblastoma cells to oxidative stress-induced cell death by attenuating PP2A-B55α-mediated inactivation of MAPKAPK2-RIPK1 signaling
Source: Cell Death Discov. 2023 Jul 27;9:265. doi: 10.1038/s41420-023-01572-1 (PMC10374899; doi:10.1038/s41420-023-01572-1)
Supplement: Supplementary file 1 — Supp Material - Tables and Figures [file 41420_2023_1572_MOESM1_ESM.pdf]

Supplementary Figures

mutation	primer sequence
R271A	FW: 5' GAGTCTATAAGCAAGGCAAAAAAGGAAGATGAC 3'
	RV: 5' GTCATCTTCCTTTTTTGCCTTGCTTATAGACTC 3'
R369D	FW: 5' GTTGCCACTTTCCTGATCGACCACAGGTTTGCAGAACC 3'
	RV: 5' GGTTCTGCAAACCTGTGGTCGATCAGGAAAGTGGCAAC 3'

Table S1. List of primers used for site-directed mutagenesis of PME-1

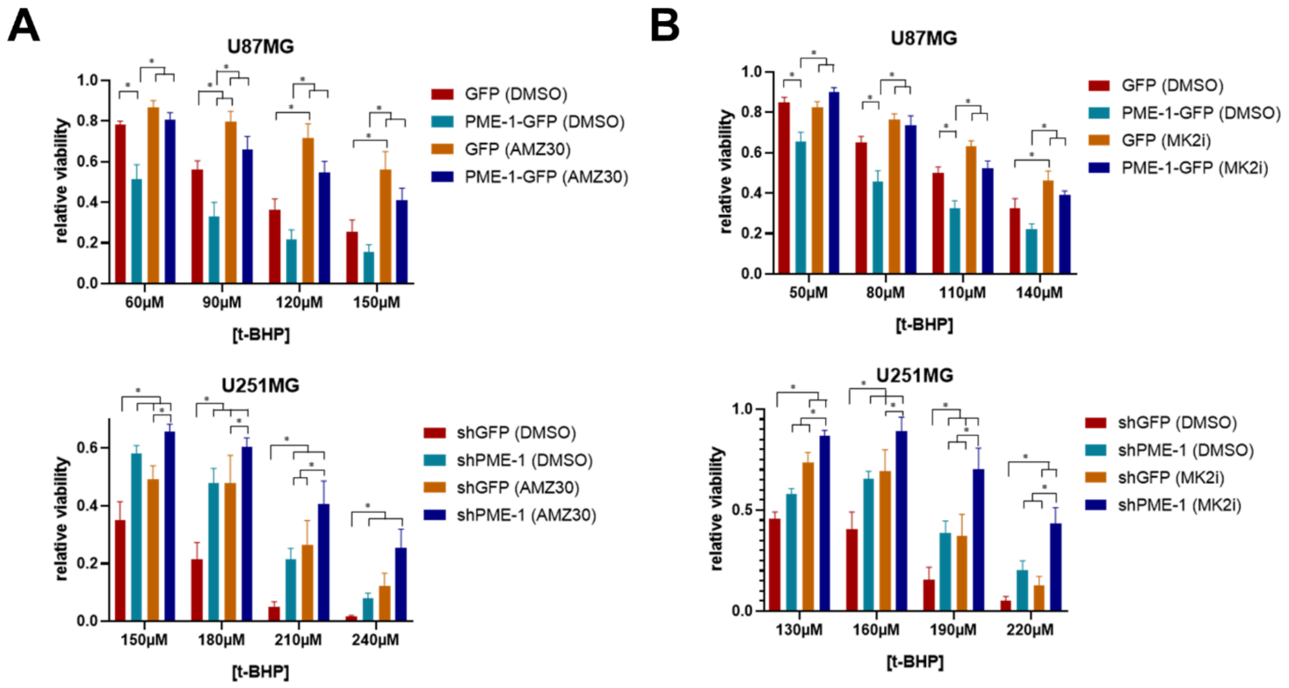

Figure S1. The PME-1 inhibitor AMZ30 and a MAPKAPK2 inhibitor both induce resistance to t-BHP. A, Sensitivity of U87MG PME-1-GFP and U251MG shPME-1 cells to 16h t-BHP treatment (different concentrations) with or without pretreatment with 20 µM AMZ30 was determined using MTT (same data as in **Fig. 2E**, but including statistical significance, N=4 (U87MG), N=6 (U251MG), two-way ANOVA, mean plus SEM). *B*, Sensitivity of U87MG PME-1-GFP and U251MG shPME-1 to 16h t-BHP treatment (different concentrations) with or without 1h pretreatment with 10 µM MK2i was determined using MTT (same data as in **Fig. 4F**, but including statistical significance, N=4 (U87MG), N=6 (U251MG), two-way ANOVA, mean plus SEM).

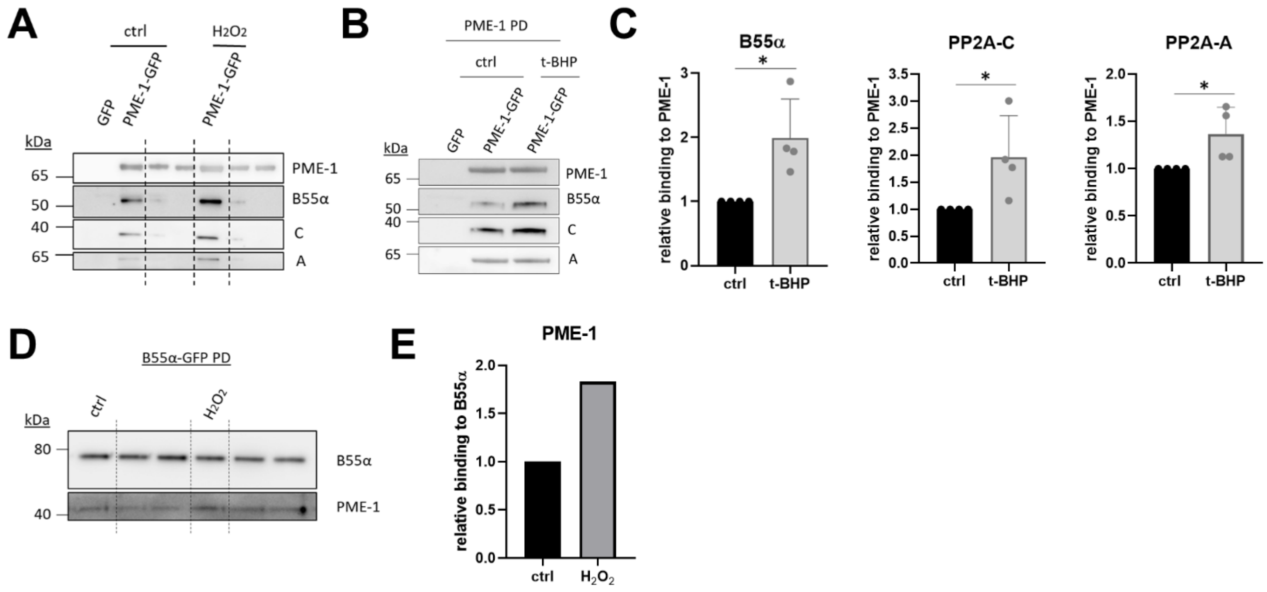

Figure S2. Oxidative stress promotes B55α nuclear localization and PP2A-B55α binding to PME-1. *A*, Non-cropped blot from **Fig. 3F**. *B*, B55α, PP2A-C and -A subunit binding to PME-1-GFP in U87MG cells was determined after 1h treatment with 1 mM t-BHP using Western blotting. *C*, Quantification of B55α, PP2A-C and -A subunit binding to PME-1-GFP shown in (B) (N=4, one-way ANOVA). *D*, PME-1 binding to B55α-GFP in U87MG cells was determined after 1h treatment with 1mM H_2O_2 by GFP pull down followed by Western blotting. *E*, Quantification of PME-1 binding with B55α-GFP shown in **D** (N=1).

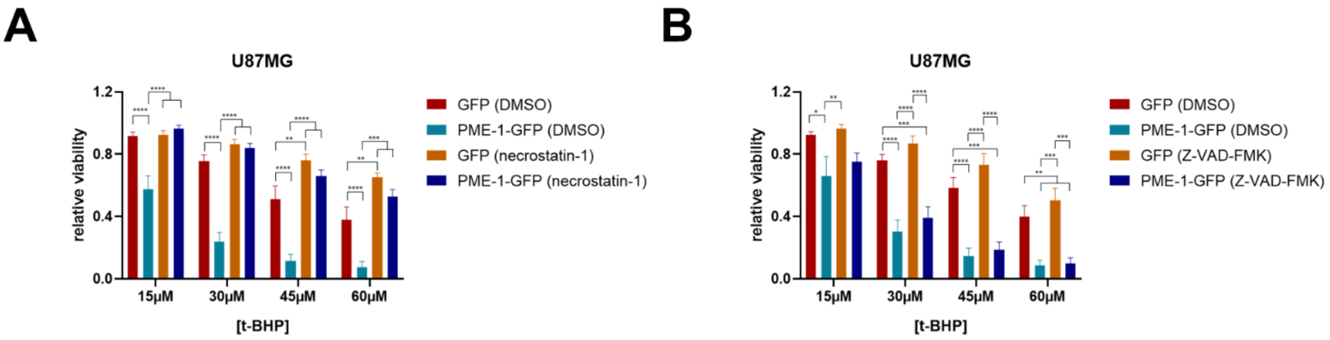

Figure S3. The increased sensitivity of PME-1 overexpressing cells to oxidative stress is RIPK1 activity dependent and caspase independent *A*, Sensitivity of U87MG PME-1-GFP to 16h t-BHP treatment (different concentrations) with or without 8h pretreatment with 100 μM necrostatin-1 was determined using MTT (same data as in **Fig. 5C**, but including statistical analysis, N=4, two-way ANOVA, mean plus SEM). B, Sensitivity of U87MG PME-1-GFP to 16h t-BHP treatment (different concentrations) with or without 8h pretreatment with 100 μM Z-VAD-FMK was determined using MTT (same data as in **Fig. 5D**, but including statistical analysis, N=4, two-way ANOVA, mean plus SEM).
