# Supplementary material for: PME-1 sensitizes glioblastoma cells to oxidative stress-induced cell death by attenuating PP2A-B55α-mediated inactivation of MAPKAPK2-RIPK1 signaling
Source: Cell Death Discov. 2023 Jul 27;9:265. doi: 10.1038/s41420-023-01572-1 (PMC10374899; doi:10.1038/s41420-023-01572-1)

Original western blots

Uncropped blots related to Figure 1

Fig.1A

VINCULIN

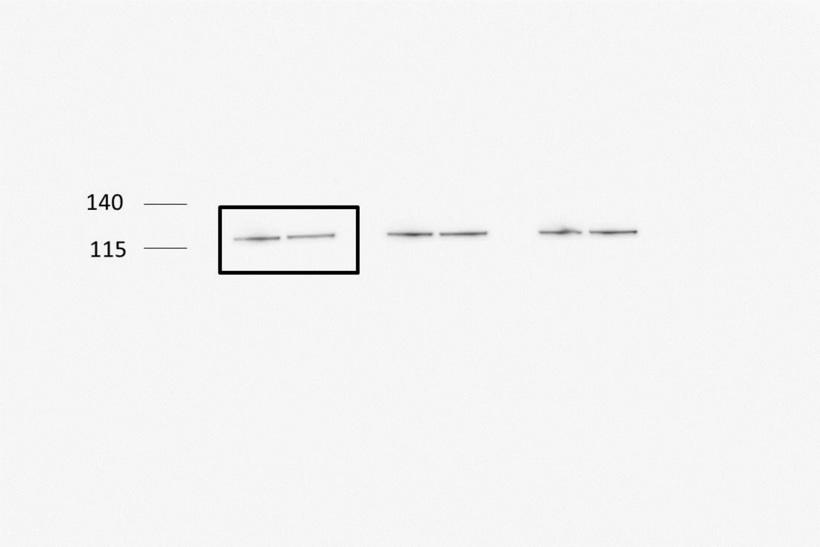

PME-1

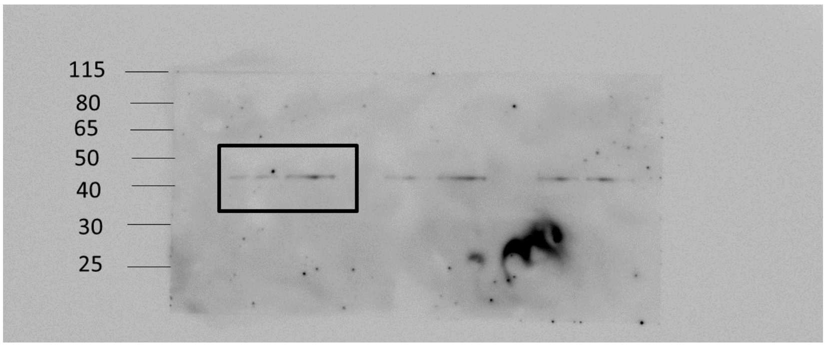

Fig.1C

VINCULIN

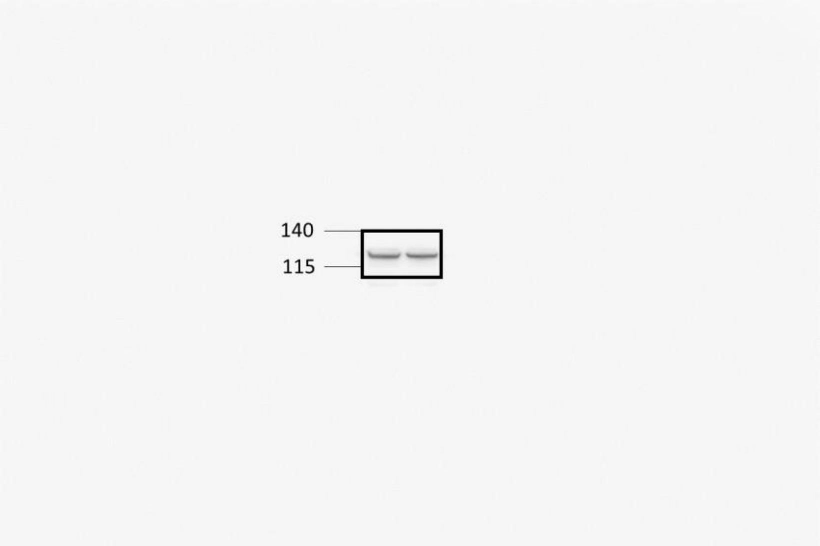

PME-1

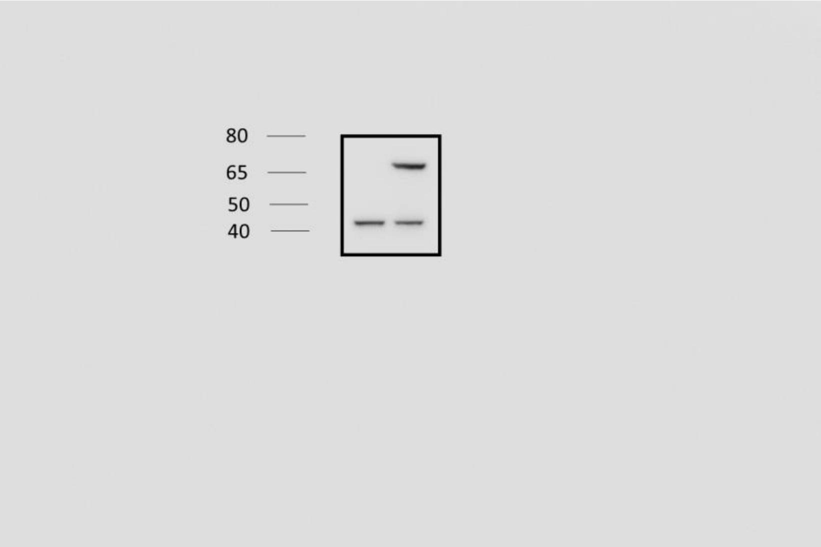

Fig.1D

VINCULIN

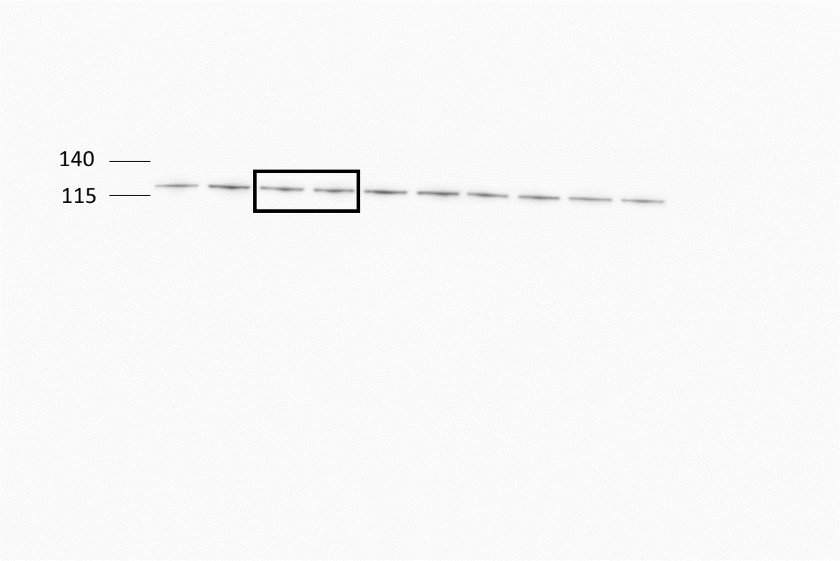

PME-1

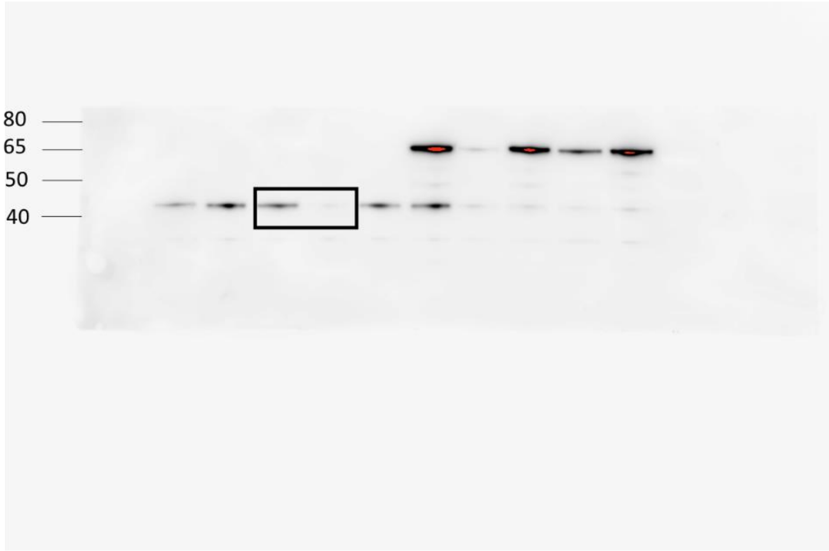

Uncropped blots related to Figure 2

Fig.2D

VINCULIN

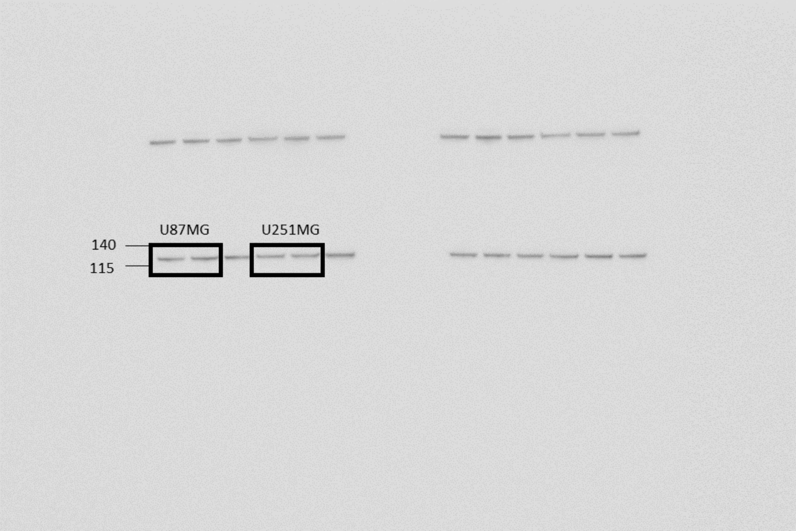

C

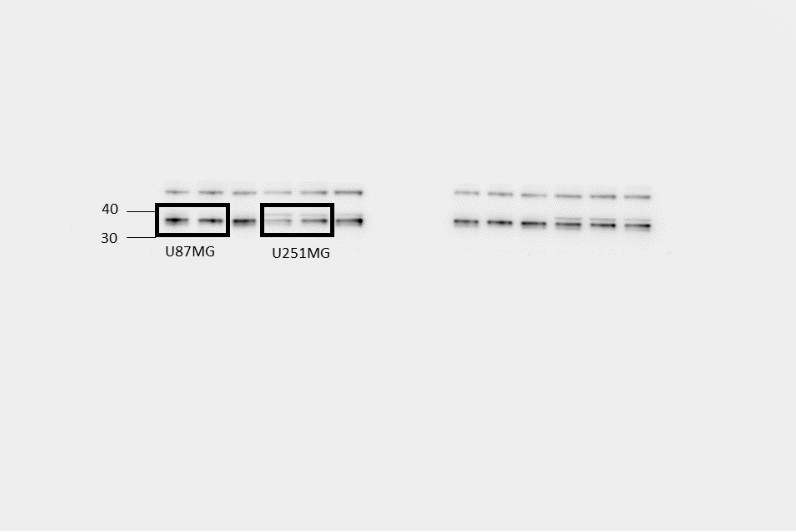

VINCULIN

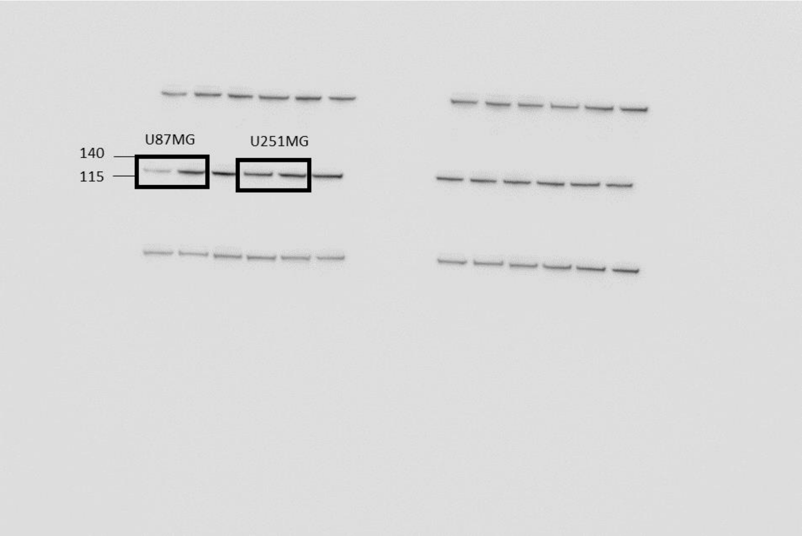

DEM C

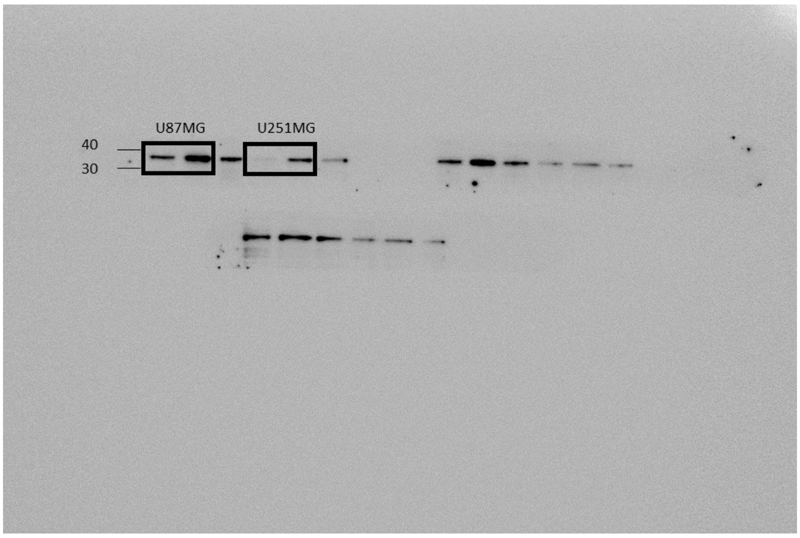

Uncropped blots related to Figure 3

Fig.3A

PONCEAU

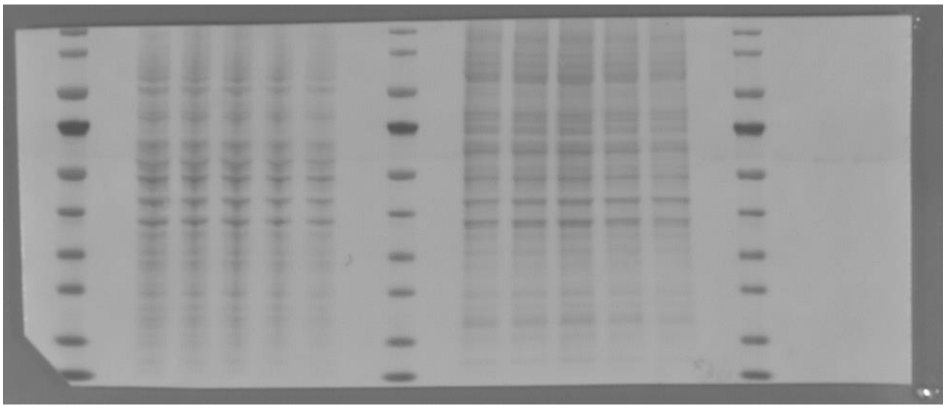

HSP90

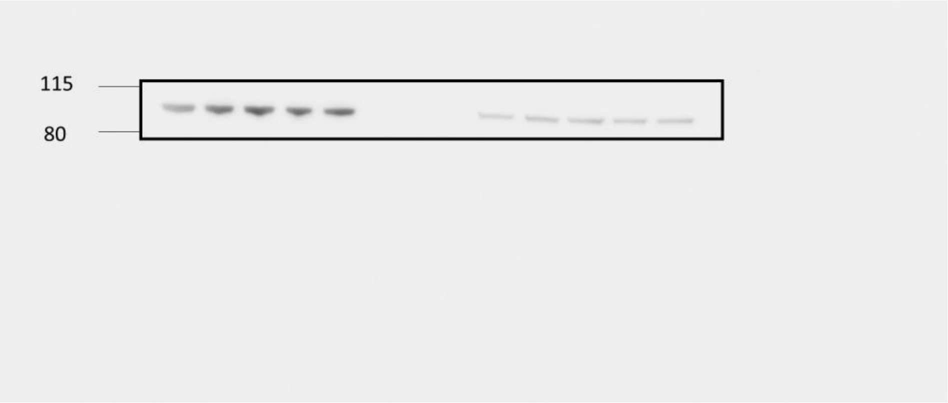

PME-1

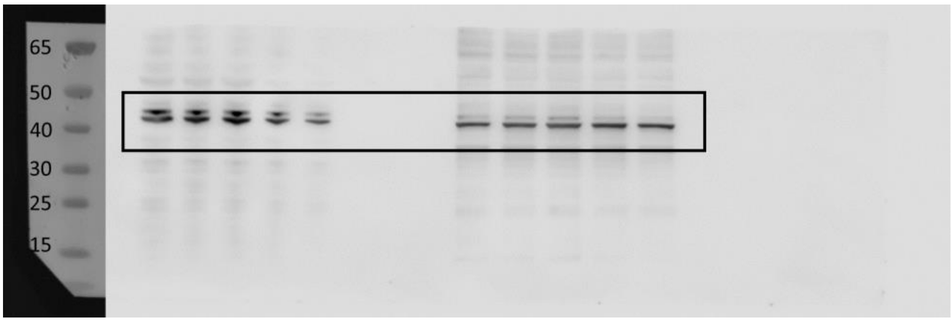

B55α

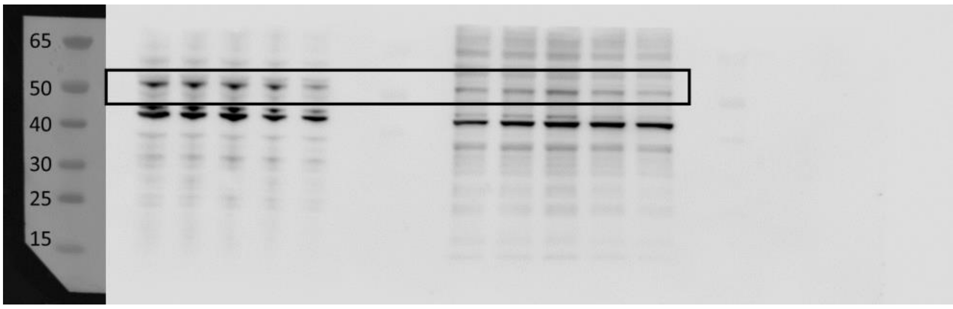

PONCEAU

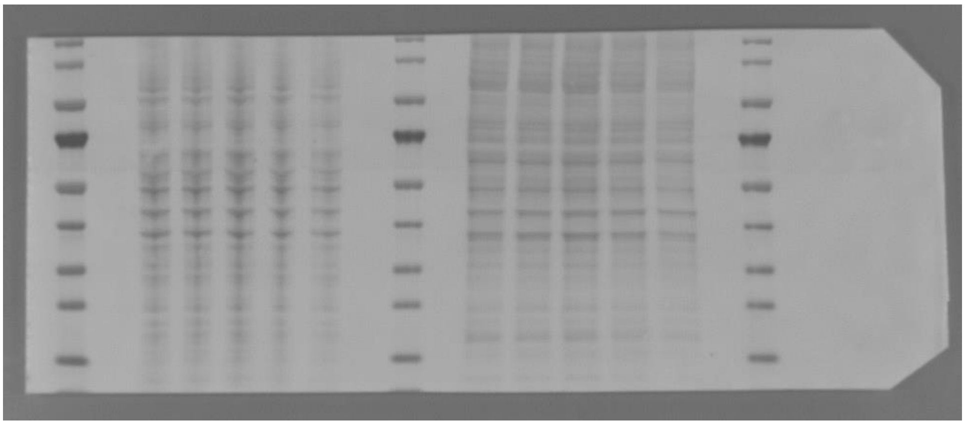

SP1

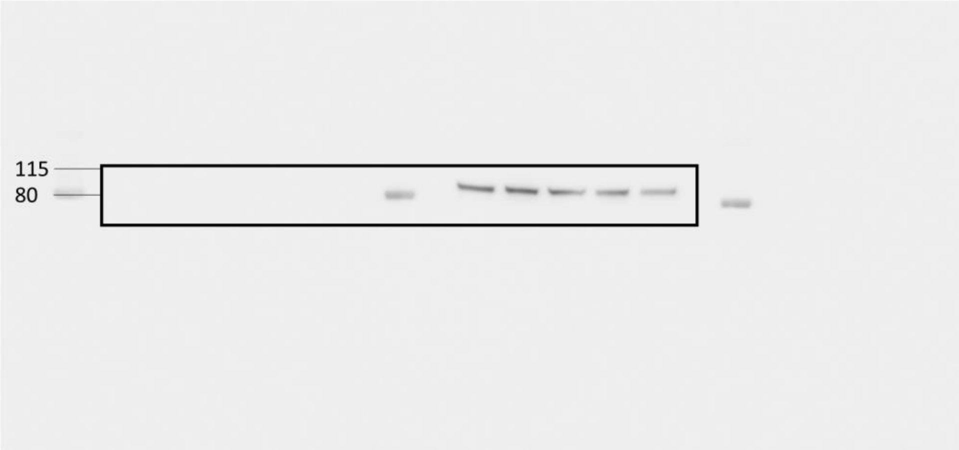

A

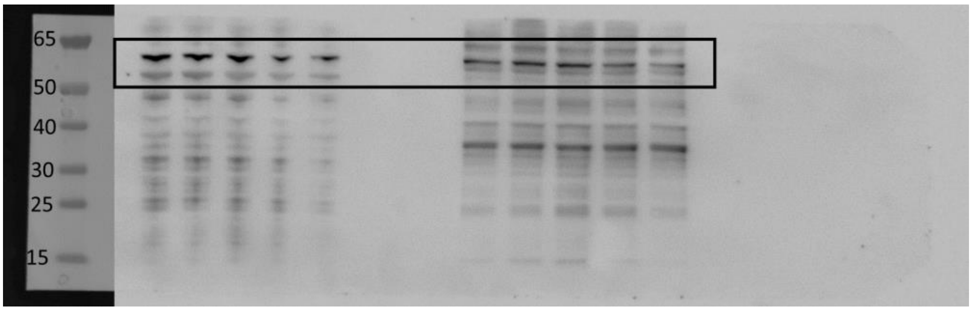

C

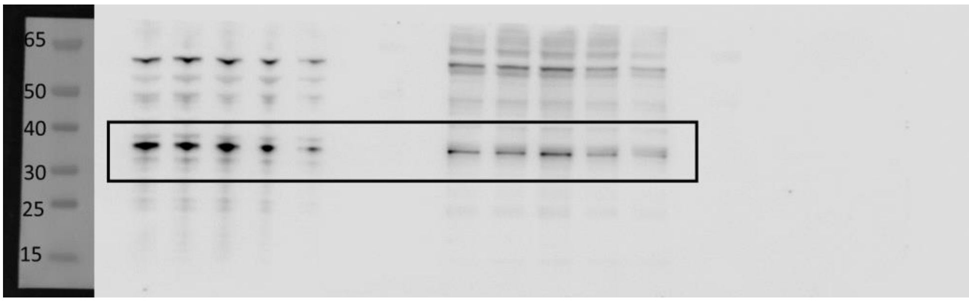

Fig.3D

VINCULIN

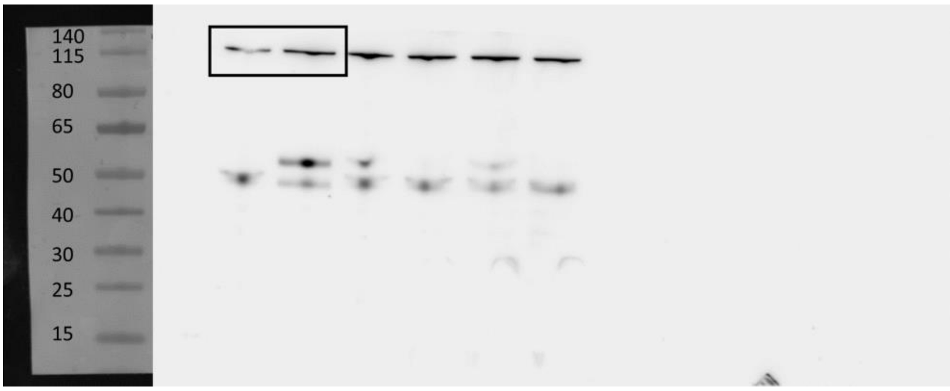

B55α

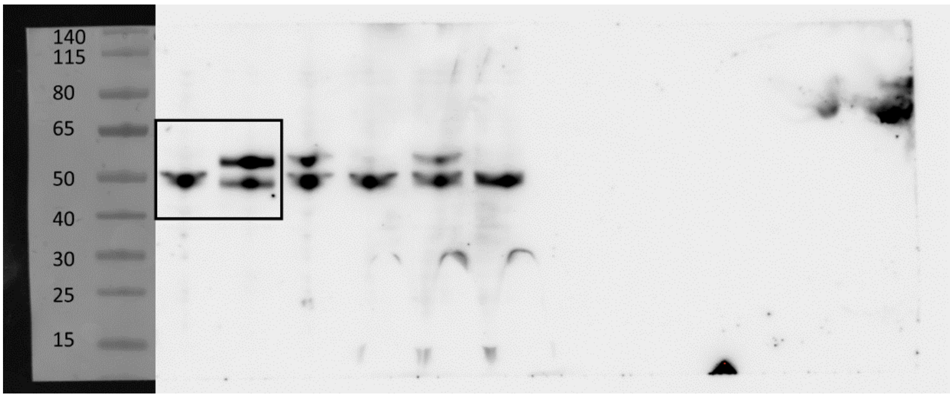

Fig.3F (same as Fig.S2A)

PME-1

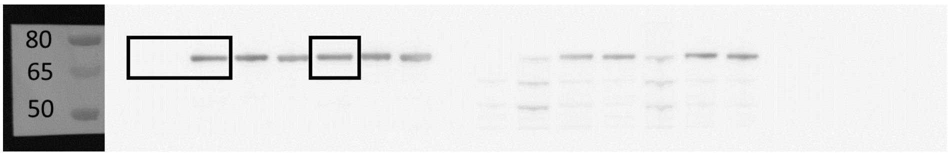

B55α

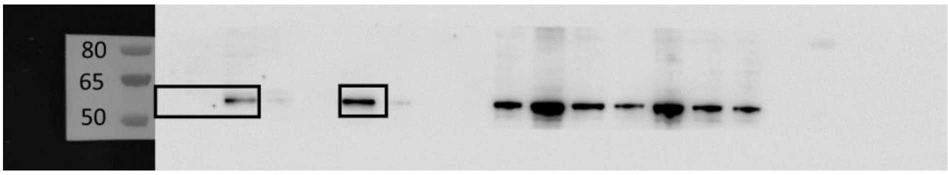

C

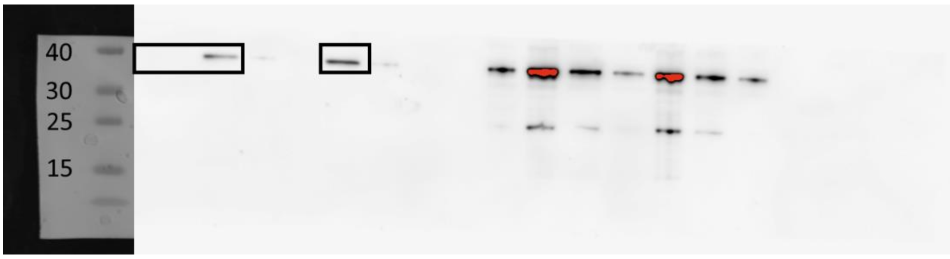

A

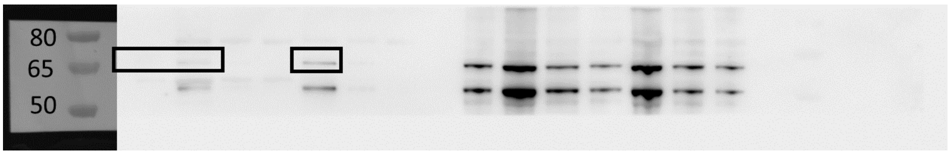

Fig.3H

B55α

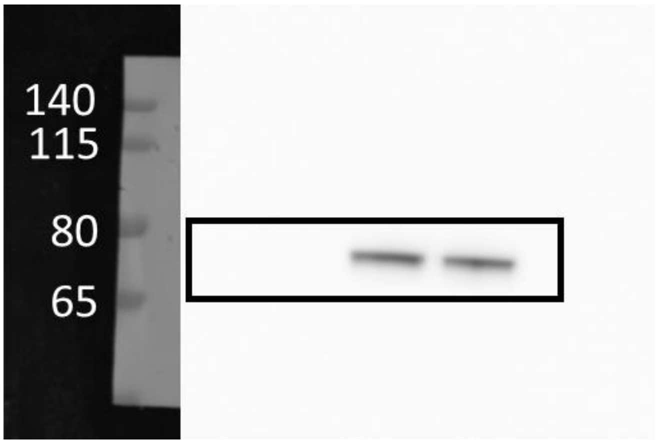

C

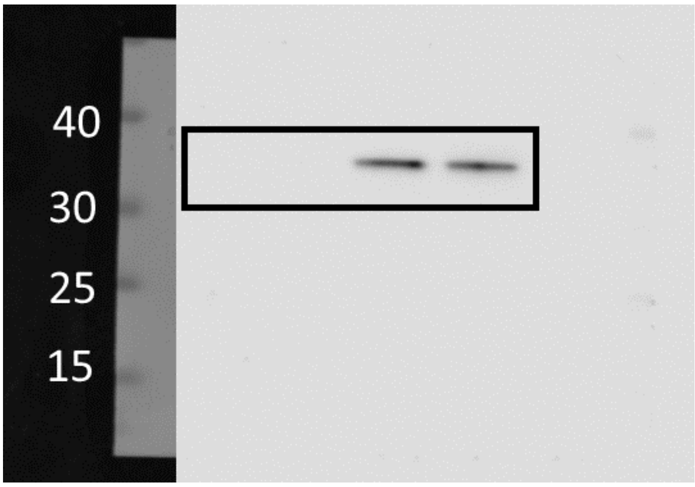

A

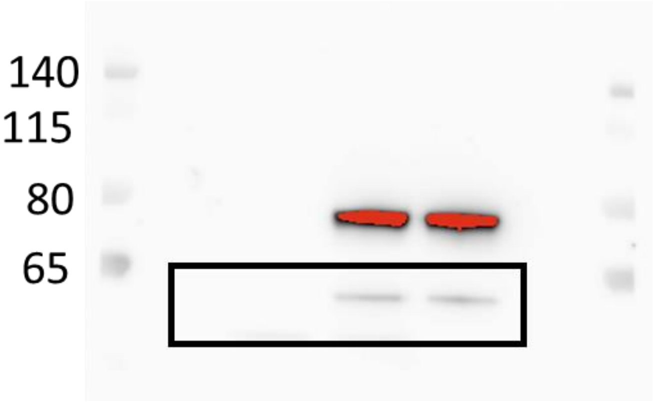

PME-1

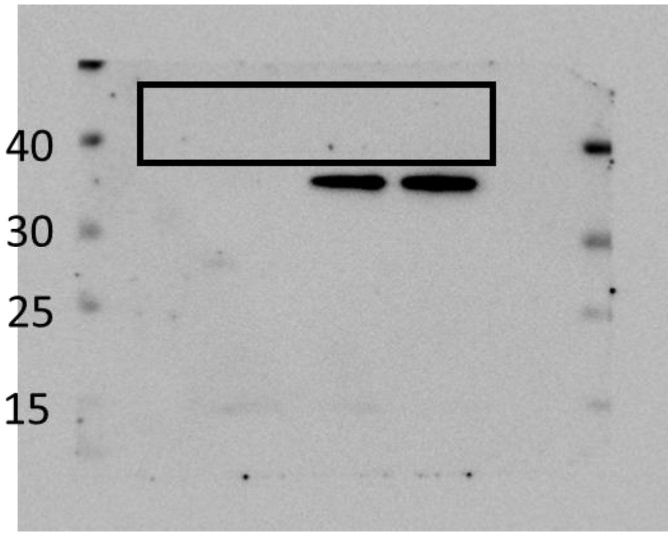

B55α

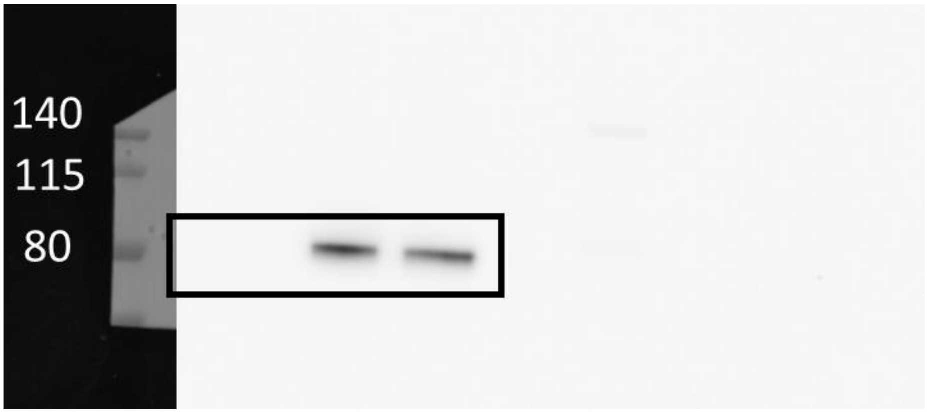

DEMC

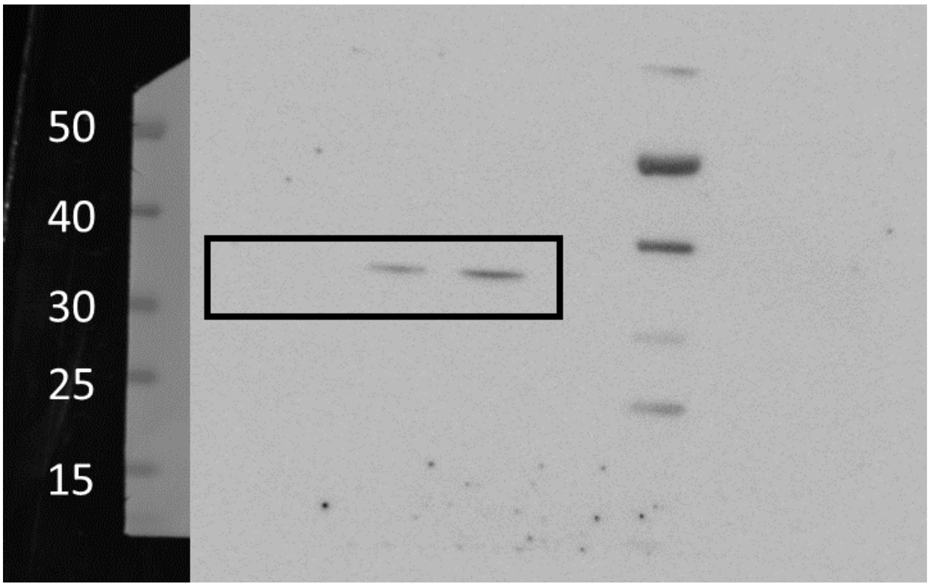

Uncropped blots related to Figure 4

Fig.4A

PONCEAU

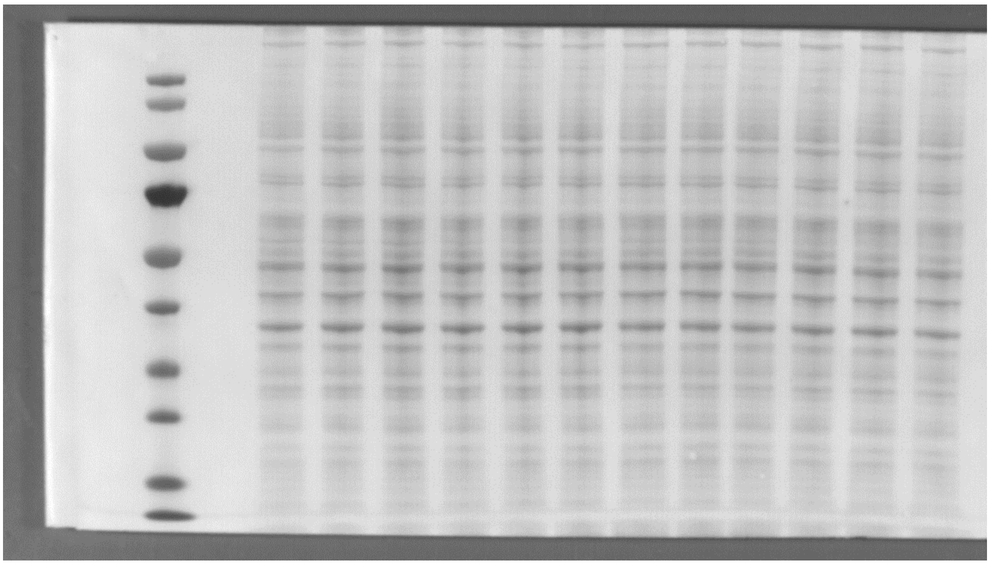

P-MAPKAPK2

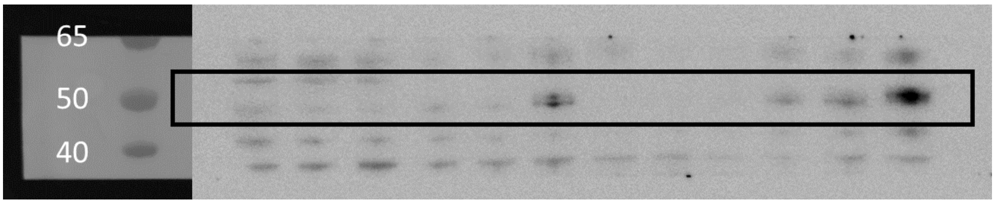

PONCEAU

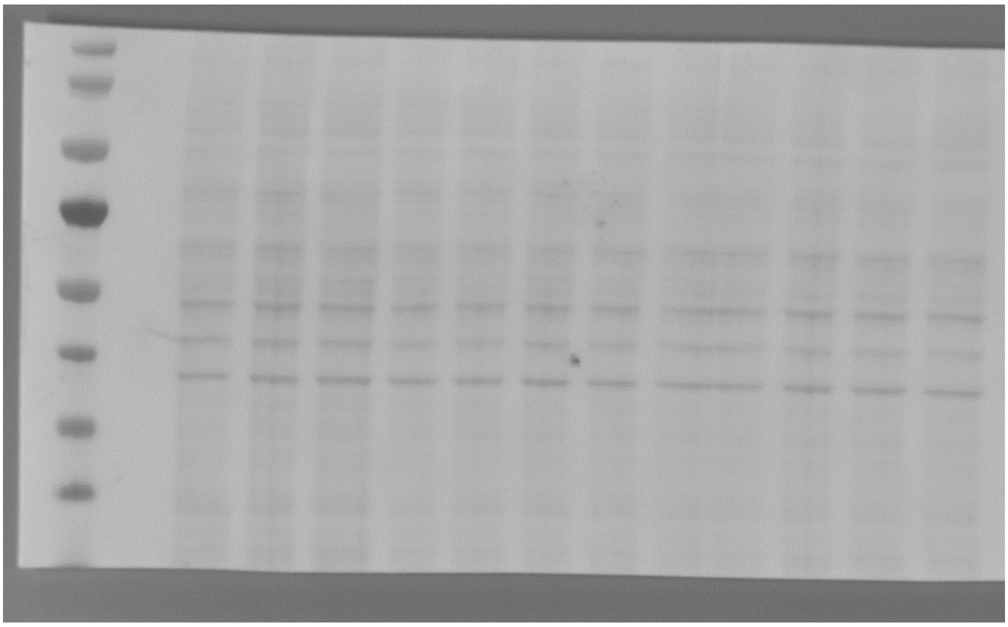

MAPKAPK2

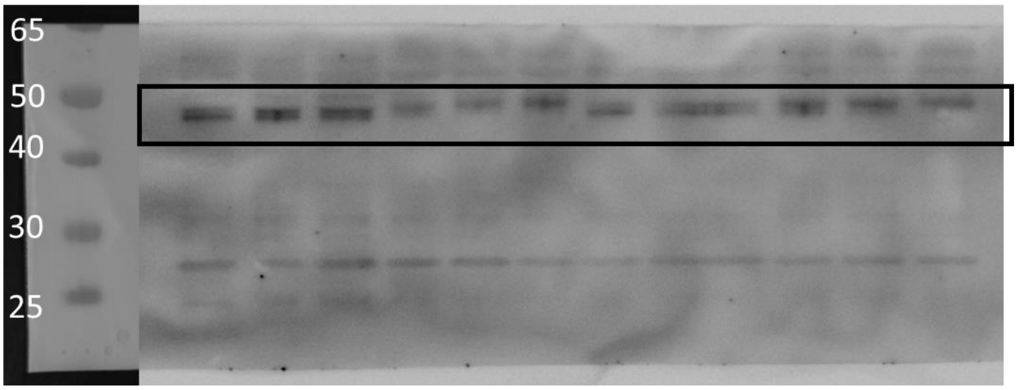

Fig.4C

PONCEAU

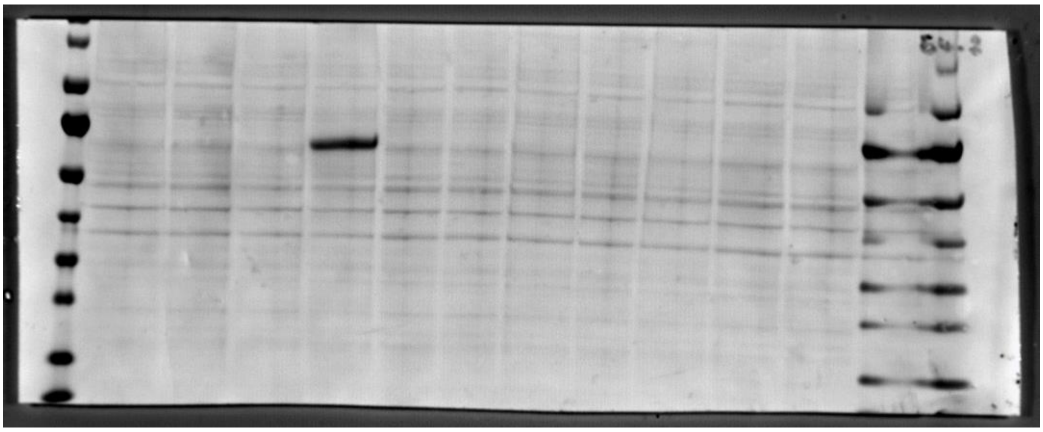

P-MAPKAPK2

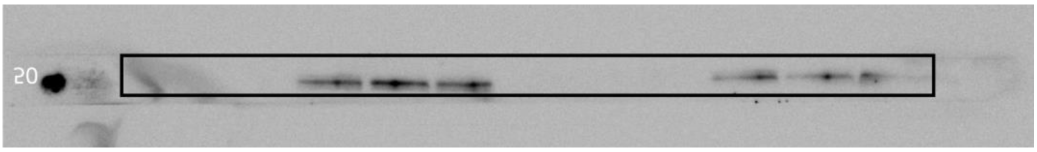

PONCEAU

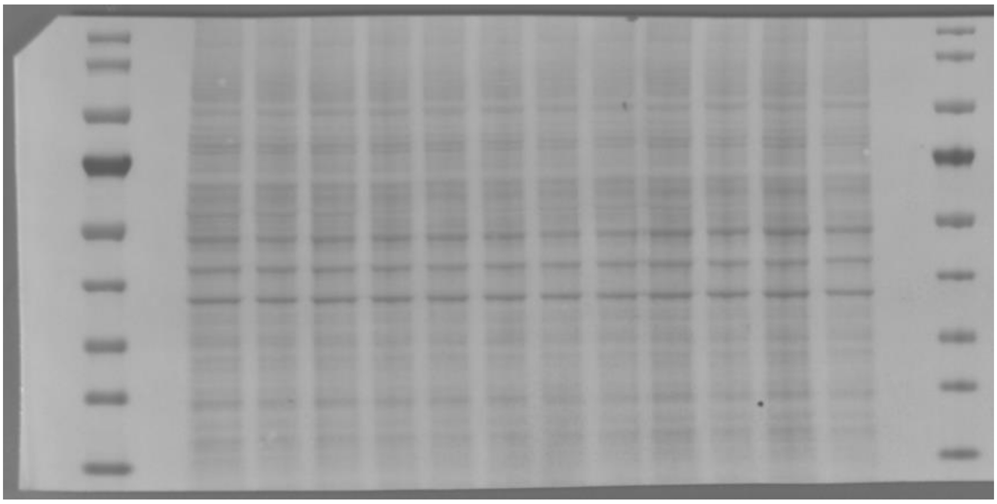

MAPKAPK2

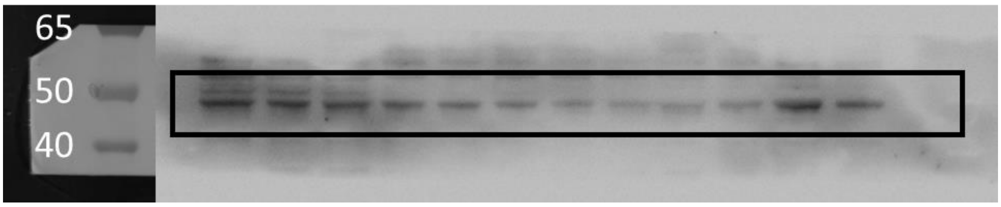

Uncropped blots related to Figure 5

Fig.5A

PONCEAU

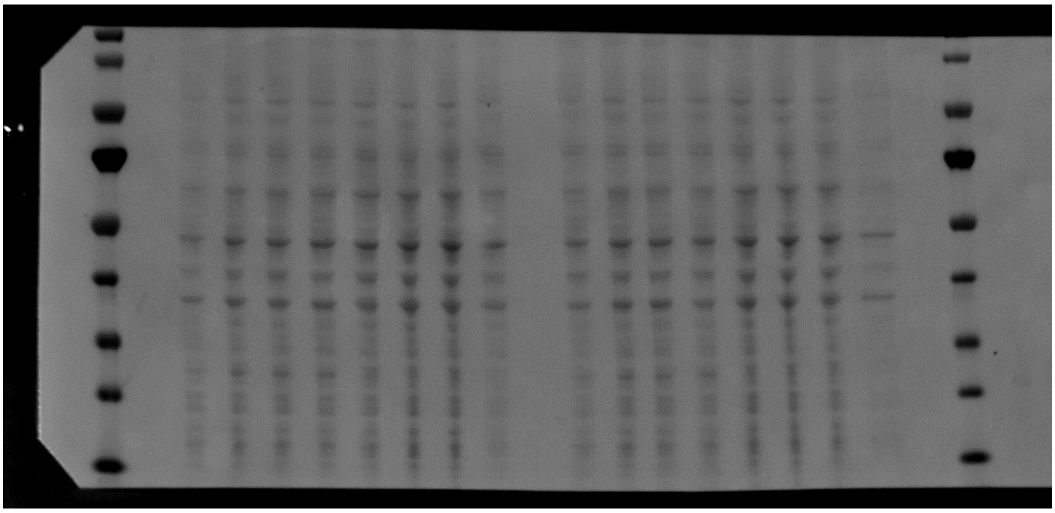

P-RIPK S320

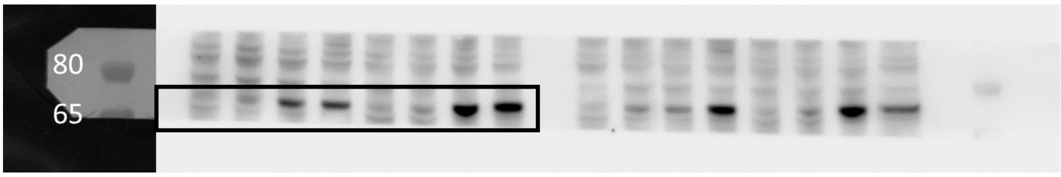

PONCEAU

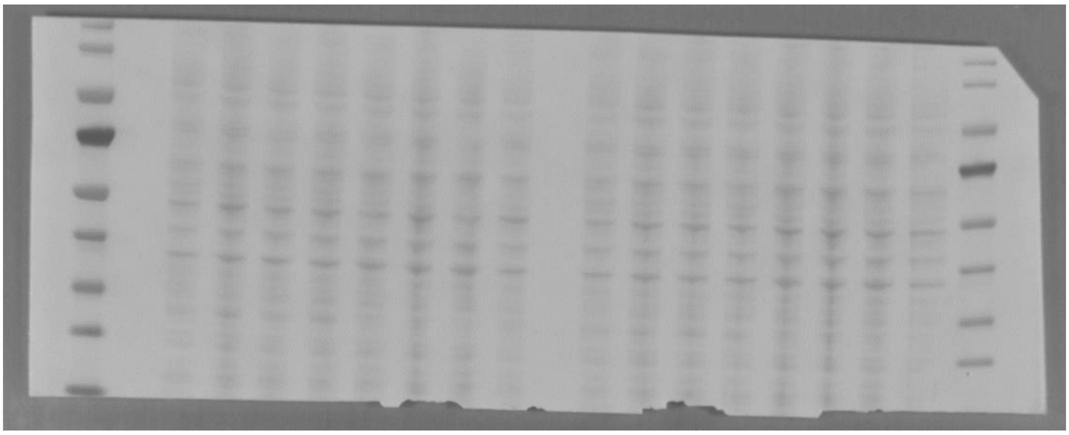

RIPK

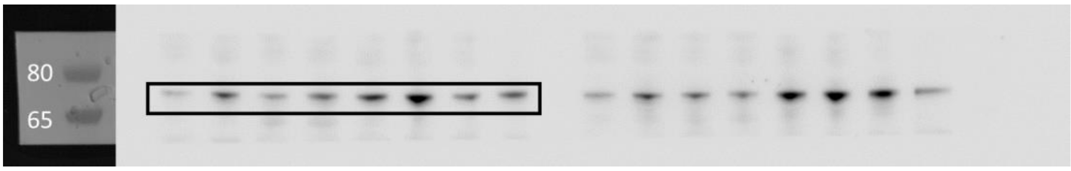

Uncropped blots related to Figure S2

Fig.S2A (same as Fig.3F)

Fig.S2B

PME-1

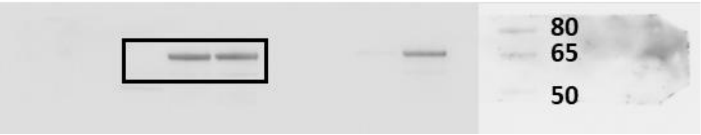

B55α

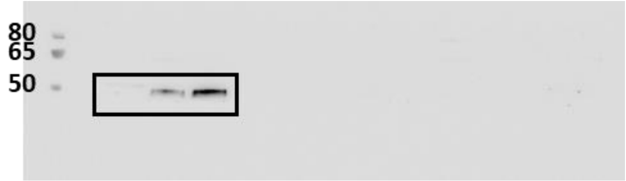

C

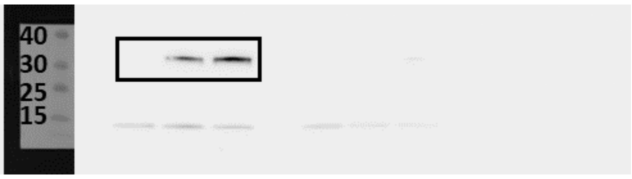

A

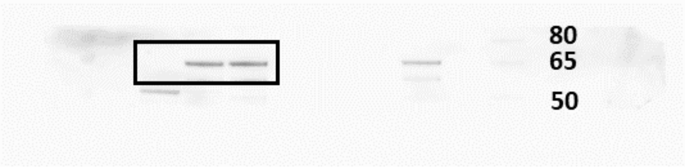

Fig. S2E

B55α

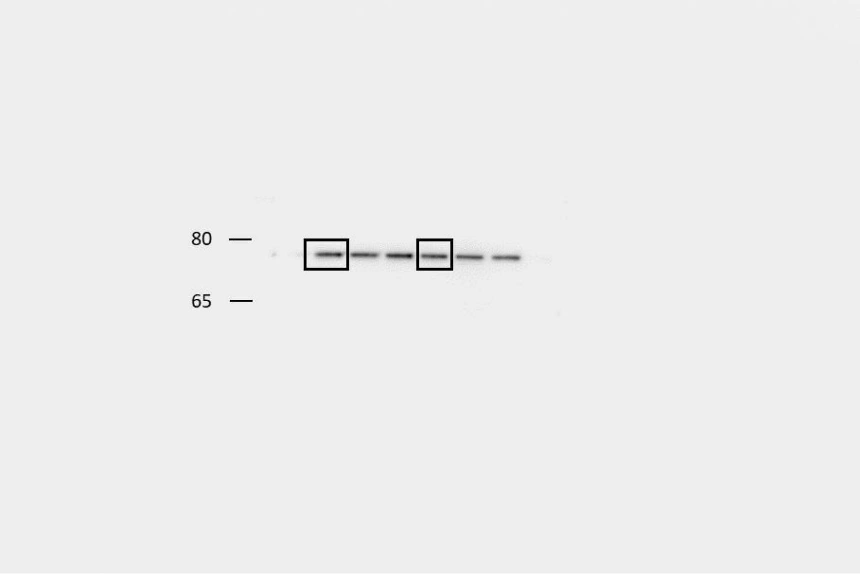

PME-1

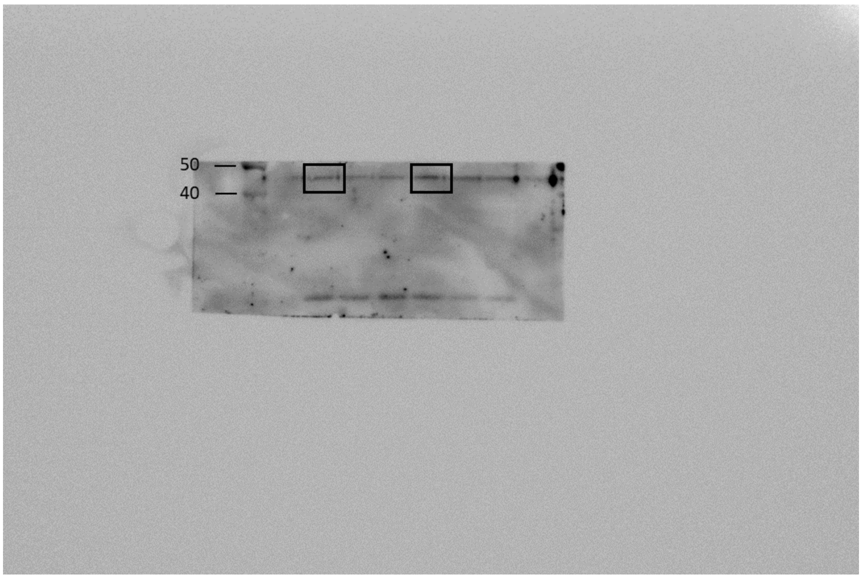

Supplement: Supplementary file 2 — Original Data File [file 41420_2023_1572_MOESM2_ESM.pdf]
